# Reinforcement Learning for Molecule Design: A Taxonomy of Algorithms, Benchmarks, and Road to Synthesizable Leads

**DOI:** 10.21203/rs.3.rs-11184036/v1

**Published:** 2026-10-16

**Authors:** Kusal Debnath, Taksheel Shetty, Preetam Ghosh

## Abstract

Reinforcement Learning (RL) has been in the forefront in traversing the vast chemical space for novel drug discovery. In this survey, we discuss the landscape of applying RL in two related paradigms: de novo drug design, the process of generating novel drugs from scratch and drug structure optimization, an approach to enhance the drug-likeness of an existing drug, with a case study for the latter.We describe the field along a taxonomy spanning value-based, policy-gradient, reward-proportional, and search-based methods, highlighting how molecular representation methods have evolved from pioneering RNN-based approaches to the latest transformer-based language models. Language models are treated as a cross-cutting layer across these families, most recently as agents that acton a molecule through chemical tools. We critically assess the frequently used datasets for training the models and benchmarks to evaluate the performance of those models across various drug generation and structure optimization tasks, arguing that strong benchmark performance does not always translate into real-world utility. We also showcase the recurrent challenges in modeling drugdesign algorithms and possible mitigation, with a final discussion on the overall developmental landscape in this field with future prospects.

**ORCID:** Kusal Debnath, 0000-0002-9389-380X; Preetam Ghosh, 0000-0003-3880-5886.

## 1 Introduction

Novel drug discovery can be defined as a search problem conducted over an astronomically large chemical space. The number of synthetically feasible, drug-like small molecules is estimated to be over $10^{60}$(Carlsson and Luttens 2024). Recent generative sampling approaches like GDB-20(Buehler et al. 2026), even though cataloged the largest set of drug-like molecules till date with an order of $10^{13}$, still covers only a vanishing fraction of the estimated space. Public repositories hosting experimentally validated compounds (e.g., ChEMBL(Zdrazil et al. 2024), PubChem(Kim et al. 2025b), BindingDB(Liu et al. 2025)) are also smaller by many orders of magnitude. This mismatch between the vastness of accessible chemistry and the sparsity of validated data is further exacerbated by the multi-objective nature of the problem: a viable candidate must simultaneously satisfy multiple criteria governed by noisy and frequently conflicting oracles.

Traditional high-throughput and combinatorial screening methods traversing this landscape are slow and costly, necessitating computational strategies that can propose efficient and practical in silico molecule design. Evolutionary algorithm-based approaches(Glen and Payne 1995) pioneered the automated, constraint-guided generation of molecules by framing molecule generation as a search-and-construction problem. Later, particle-swarm-based combinatorial search(Hartenfeller et al. 2008) offered a complementary population-oriented route for fragment-based de novo drug design. Prior to the adoption of deep learning, these search-based paradigms explored chemical space without relying on a learned molecular representation.

The rise of deep learning subsequently reshaped the field with pivotal approaches. Recurrent Neural Network (RNN)-based methods guided the path for SMILES-based molecular generation(Segler et al. 2018). Later, Variational Autoencoder (VAE)-based models introduced a continuous latent space amenable to gradient-based optimization(Gómez-Bombarelli et al. 2018), relying solely on the distributional similarity within the training data. Generative Adversarial Networks (GANs)(Kadurin et al. 2017) offered a sound alternative for navigating chemical space through adversarial training. Nevertheless, in all the generative scenarios above, a directed, property-specific feedback signal remained absent.

RL-based approaches entered the space as a bridge to transform these generators into goal-directed optimizers. Rather than only reproducing the statistics of a training corpus, an RL-tuned generator receives a scalar reward for each molecule it proposes and updates its policy to increase the frequency of high-reward structures(Sutton and Barto 1998). In practice, instead of disregarding the merits of the previously mentioned pioneer methods, RL mostly acts as an additional layer on top of a pre-established generative backbone where any of those supervised algorithms acts as a prior to a later RL-guided fine-tuning stage. The prior learns the syntactic and semantic rules of generating synthetically viable molecules which are further fine-tuned based on specific-objectives for a goal-directed molecule design landscape. In this survey, we keep the scope of molecule design limited to small molecules only, as it is the most widely investigated class of molecules in drug design.

Goal-directed molecule design can be bifurcated into two streams: de novo molecule generation and molecule optimization. De novo molecule generation approaches discuss designing a desired molecule from scratch(Sumita et al. 2026). On the other hand, molecule optimization, also known as lead optimization, is the process of modifying the structure of an existing lead structure to optimize its drug-like properties(Zhang et al. 2024a). Both these streams are useful in different yet complementary scenarios. A de novo molecule generation workflow is needed to generate a library of novel drugs to target undruggable protein targets, where a molecule optimization pipeline is used to improve the efficacy of an existing drug available in the market through redesigning its core functional components.

Several prior surveys exist on generative and RL-assisted molecule design. Nevertheless, such surveys are with critical research gaps. First, no prior review compares evaluation benchmarks based on whether they take a user-supplied lead as input or whether the structural modifications performed are grounded in real reaction chemistry. Broad generative reviews organise benchmarks by architecture or by metric (Elton et al. 2019; Bilodeau et al. 2022; Du et al. 2024), while a benchmark-centric review predates the current suites (Grant and Sit 2021). Surveys on lead optimisation indicates towards the absence of input-conditioned benchmarks (Zhang et al. 2024b; Xia et al. 2024). We therefore classify benchmarks by permitted oracle class, budget cap, and a lead input flag. Second, no prior review stratifies the reward signal itself. Existing reviews enumerate oracles as properties or as scoring functions. We instead organise the oracles into four tiers ordered by fidelity and cost per call: heuristic descriptors, learned QSAR classifiers, docking, and physics-based free-energy methods (Figure 3). Third, almost no survey performs computational analysis. Only two of the eleven have any empirical component: a meta-analysis of metrics reported by others (Flores-Hernandez and Martinez-Ledesma 2024) and a partial reproduction of three optimisers under matched oracle budgets (Xia et al. 2025). Neither executes an input-conditioned lead-optimisation study. We perform a comprehensive case study on four deconstructed DRD2 recognition cores plus risperidone as a negative control defining the reward ceiling, optimised for the quantitative estimate of drug-likeness (QED), synthetic accessibility (SA) and DRD2 activity with explicit synthesis routes. To our knowledge, this is also the only survey to treat RL-driven de novo generation and RL-driven lead optimisation as one taxonomy, while restricting scope to small molecules. Table 1 shows the scope of the prior surveys, their limitations and how our work succeeds by addressing those limitations.

In summary, we aimed to seek answers for the following research questions in the space of RL-driven molecule design:

- RQ1: How do RL algorithm families for small-molecule design differ in the exploration–exploitation trade-offs they make, and what does that imply for lead optimisation?
- RQ2: What reward oracles are used, and how does oracle fidelity and cost per call constrain the achievable sample efficiency?

**Table 1**: Positioning of this survey against prior reviews of generative and RL-based small-molecule design. *Lit. window* is the span of literature reviewed; entries marked ≈ are inferred from the newest reference year because the survey does not state a window. *Data/Bench.* reports whether a dedicated treatment of training datasets (D) and of standardised evaluation benchmarks (B) is present. *Corpus* gives papers reviewed where stated, otherwise the reference count. Abbreviations: MolOpt = molecule (lead) optimisation, LM = language model, PG = policy gradient

| **Survey** | **Scope** | **Lit. window** | **Primary taxonomy axis** | **Data/Bench.** | **Empirical component** | **Corpus** |
|---|---|---|---|---|---|---|
| Elton et al. (Elton et al. 2019) *Mol. Syst. Des. Eng.* | RNN, AE/VAE, GAN generators; RL as one method family; SMILES/graph/3D; molecules and materials | ≈2016–2019 | Representation → architecture → reward/metric | D: yes (tables); B: partial (MOSES, GuacaMol) | None | 45 papers |
| Grant & Sit (Grant and Sit 2021) *RSC Med. Chem.* | Benchmarking of de novo generators (FCD, GuacaMol, MOSES) | ≈up to 2021 | By benchmark platform | D: no; B: yes | None | 40 refs |
| Bilodeau et al. (Bilodeau et al. 2022) *WIREs Comput. Mol. Sci.* | 1D/2D/3D generative models; RL, Bayesian Optimisation (BO) and Genetic Algorithms (GA) treated as interchangeable optimisers | ≈up to 2021 | Design choice → problem statement → benchmark | D: partial; B: yes (MOSES, GuacaMol) | None | 118 refs |
| Tang et al. (Tang et al. 2024) *Brief. Bioinform.* | Diffusion-centric generative AI; small molecules *and* proteins; RL absent | ≈up to 2023 | Application theme → subtask | D: yes (per subtask); B: no standard suite | Compiles self-reported numbers; no runs | ≈158 refs |
| Du et al. (Du et al. 2024) *Nat. Mach. Intell.* | VAE/GAN/AR/flow/ diffusion/EBM/GFlowNet; RL, GA, MCTS, BO as optimisers | ≈up to 2024 | Task → design decision → evaluation | D: no section; B: partial (GuacaMol, MOSES, PMO) | None; curated table of wet-lab-validated cases | 173 refs |
| Xia et al. (Xia et al. 2024) *Quant. Biol.* | MolOpt only: sequence/graph mapping, distribution matching, guided search | ≈up to 2023 | Optimisation methodology class | D: partial; B: no standard suite | Compiled comparisons on paired datasets; no runs | 93 refs |
| Zhang et al. (Zhang et al. 2024b) *arXiv* | Lead optimisation as constrained subgraph generation; 2D and pocket-aware | ≈up to 2024 | MolOpt subtask by input/output (linker, scaffold hop, side-chain, fragment swap) | D/B: none; argues MolOpt benchmarks do not exist | None | 32 methods; 101 refs |
| Flores-Hernandez & Martinez-Ledesma (Flores-Hernandez and Martinez-Ledesma 2024) *J. Cheminform.* | Chemical LMs (RNN, transformer, VAE, GAN, S4) plus biasing (TL, RL, conditional) | Jan 2020–Jun 2024 (stated) | Architecture × biasing strategy | D: yes; B: MOSES/ GuacaMol as inclusion criterion | Yes: statistical meta-analysis of reported metrics | 72 articles |
| Wang et al. (Wang et al. 2025c) *arXiv* | LLMs (≥1B params) for text-guided generation and editing; prompting, SFT, preference tuning | ≈up to 2025 | Task × tuning regime | D: yes; B: yes (MOSES, Dockstring, TOMG-Bench) | None | n.a. |
| Xia et al. (Xia et al. 2025) *Int. J. Mol. Sci.* | MolOpt: discrete-space (GA, RL) vs. latent-space methods | ≈up to 2025 | Search space × optimisation mechanism | D: yes; B: yes (TDC oracles, MOSES) | Yes: partial reproduction of three methods under matched oracle budgets | 112 refs |
| Sampa & Abdul Aziz (Sampa and Abdul Aziz 2026) *Health Sci. Rep.* | RL-only systematic review: PG, actor–critic, value-based, MCTS; small molecules | Jan 2017–Jan 2024 (stated) | RL component / review question | D: no; B: no section (GuacaMol, PMO in prose only) | None; framework is interpretive, not validated | 35 refs |
| **This survey** | RL-driven de novo generation *and* lead optimisation; small molecules only; value-based, PG, reward-proportional and search-based families with | 2015–Aug 2026 | **Three axes:** RL algorithm family; **oracle tier** (Tier 1–4 by fidelity and cost per call); **benchmark axes** (distribution vs. search, capped budget, | D: yes (17 datasets, §5.1); B: yes (7 suites incl. PMO, TDC, DOCKSTRING, MolScore, ChemCoTBench) | **Yes: executed** lead-optimization case study (TRACER and RGFN; four deconstructed DRD2 leads plus | 82 articles |

- RQ3: Do current benchmarks measure what lead optimisation actually requires: a modification of a supplied lead, achievable through feasible reaction chemistry?

### 2 Background

In this section, we discuss some of the most frequent terminologies and concepts related to RL-driven molecule design. We start with fundamental concepts in RL and dive deeper into concepts specific to molecule design.

#### *Reinforcement Learning*

RL is a computational framework where an agent learns to maximize the cumulative reward $G_t = \sum_{k=0}^{\infty} \gamma^k R_{t+k+1}$ through interactions within an environment(Sutton and Barto 1998). In the context of molecular design, RL can be described as a combination of three major components: an action space $A$ that comprises symbols or graph-modification operations defining the molecular structure; a policy $\pi(a|s)$ that predicts the next action $a \in A$ to extend the current partial molecule (state $s \in S$) until termination; and finally the environment that evaluates the generated molecule and assigns rewards $R(s, a)$ based on desired molecular properties.

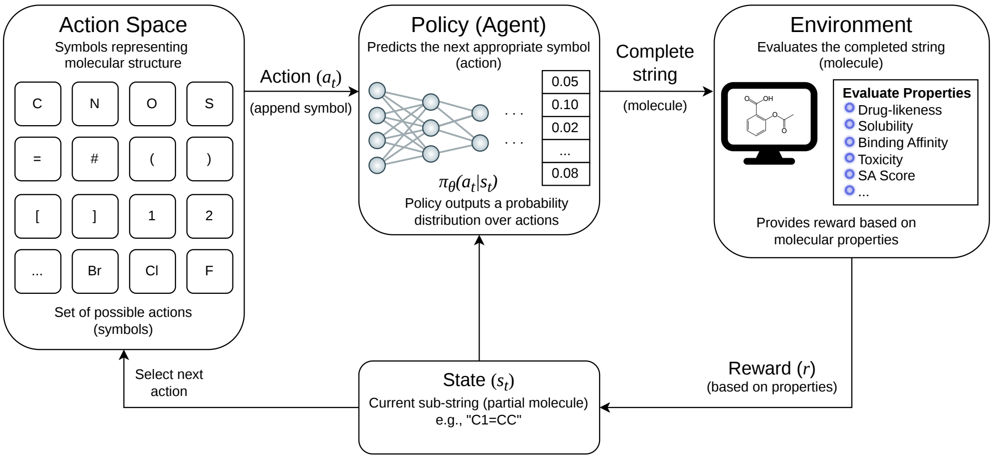

**Fig. 1**: **General workflow of an RL-based molecular design pipeline.** Based on the current state (molecule) and reward, the policy predicts the next action (symbol), chosen from the action space, to extend the current partial molecule until termination. The role of the environment is to evaluate the generated molecule and assign rewards based on its molecular properties, finally sending rewards back to the agent.

#### *Markov Decision Process*

According to the definition of Markov property(Puterman 2014), the future is independent of the past given the present, i.e., the current state $s$ must contain all information needed to make an optimal decision. The history of how the agent reached the current state $s$ does not supply any additional information.

A Markov Decision Process (MDP)(Puterman 2014) can be defined as a process for which the change of state is only dependent on the current state and independent of the past states. It is the formal mathematical framework for modeling the environment in RL. For analogy, a supervised learning problem is defined by a dataset $(X, y)$ pairs; similarly, an RL problem is defined by an MDP. Zhou et al.(Zhou et al. 2019) discussed the rationale behind formulating the modification of a molecule as an MDP.

An MDP describes the environment using several components, often represented as a tuple $(S, A, P, R, \gamma)$, defined as:

$$\mathcal{M} = (S, A, P, R, \gamma) \tag{1}$$

where:

$S$ (States) is the set of possible situations the agent can be in.

$A$ (Actions) is the set of all possible moves the agent can take.

$P$ (Transition Probability) represents the rules of the environment, i.e., the probability of ending up in a new state $s'$ after taking an action $a$ in a starting state $s$, denoted $P(s'|s, a)$:

$$P(s' \mid s, a) = \Pr(S_{t+1} = s' \mid S_t = s, A_t = a) \tag{2}$$

$R$ (Reward Function) is the feedback from the environment, i.e., the immediate numerical reward the agent gets for taking action $a$ in state $s$:

$$R(s, a) = \mathbb{E}[R_{t+1} \mid S_t = s, A_t = a] \tag{3}$$

$\gamma$ (Discount Factor) is a number between 0 and 1 that represents how much we value future rewards versus immediate rewards; $\gamma = 0$ signifies we only take the next immediate reward into consideration, while $\gamma = 0.99$ means rewards far into the future holds nearly as much value as immediate rewards. The discounted cumulative return from time step $t$ is given by:

$$G_t = \sum_{k=0}^{\infty} \gamma^k R_{t+k+1} \tag{4}$$

#### ***Policy, Value, and Search-based Paradigms***

In RL, a policy is a prescriptive attribute that decides the sequence of actions agent must take by mapping current state $s \in S$ to an action $a \in A$ (e.g., extending a partial molecule by adding an atom, bond, or fragment)(Zhou et al. 2019; Sumita et al. 2026). Policy $\pi(a|s)$ thus is stochastic in nature that provides output as a distribution over actions or a single action. A value function, on the other hand, provides a scalar output $V^\pi(s)$ or $Q^\pi(s, a)$ over an expected return from a state or state-action pair, assessing the consequences on the action, for instance, estimating how promising a partially constructed molecule is likely to be with respect to the target property. A third paradigm, search-based optimization, forgoes gradient-based policy or value updates altogether and instead treats molecule generation as a tree search over the chemical space, most commonly through Monte Carlo Tree Search (MCTS), where the search procedure itself, rather than a learned function, drives the exploration-exploitation trade-off.

#### *Generator, Evaluator and Agent*

In majority of the recent molecule design approaches, molecule design workflow consists of two major steps: the first one is typically to train a generator model, also called prior ($\pi_{\text{prior}}$). The generator learns chemically valid molecular representation and scaffold decoration rules(Fialková et al. 2021) by modeling the probability of the next token/fragment $a_t$ given the partially generated molecule (state) $s_t$, i.e., $\pi_{\text{prior}}(a_t \mid s_t)$. In the second step, the generator is followed by an evaluator that assesses the quality of the molecules generated by assigning a reward $R(s_T)$ based on the final generated molecule (terminal state $s_T$). An RL workflow usually acts as a 'messenger' between the generator and the evaluator. An agent, parameterized by a policy $\pi_\theta$ (typically initialized from $\pi_{\text{prior}}$), receives reward signal $R$ from the evaluator and steers the generator towards improved molecule generation based on that signal.

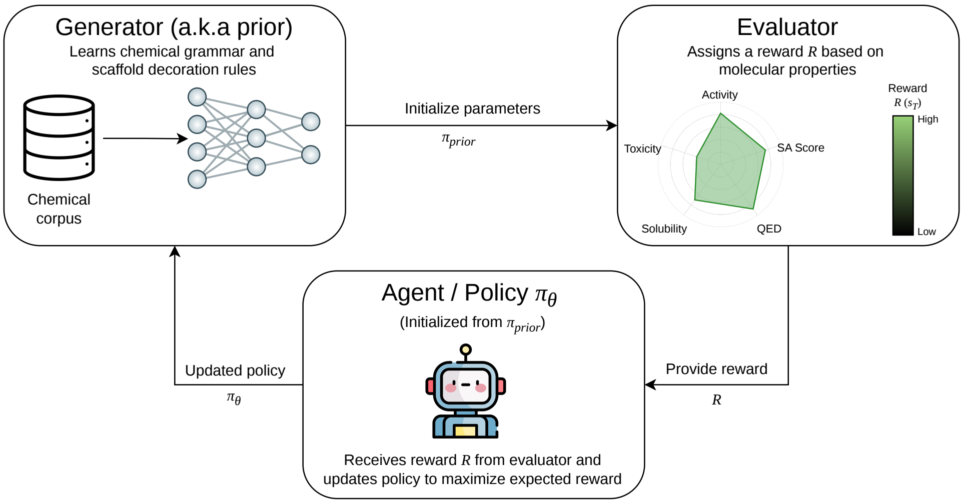

**Fig. 2**: **Generator-Evaluator workflow in molecule design.** Generator is trained to learn the rules of chemistry to be able to generate synthetically viable molecules. Evaluator assesses the specific physicochemical properties and drug-likeness of those generated molecules and assigns reward. Agent receives that reward as feedback signal and updates the policy that guides weight adjustment to the generator.

#### *Reward Design and Oracle Landscape*

The reward function is arguably the most consequential design choice in molecule design as it defines the objective the agent will pursue, including any unintended shortcuts. Oracles are the functions that convert a candidate molecule into a scalar score(Huang et al. 2021; Gao et al. 2022). Based on the fidelity and computational costs, oracles can be divided into four tiers. Tier 1 encompasses heuristic scores such as the quantitative estimate of drug-likeness (QED), the synthetic accessibility (SA) score, and penalized logP which are free ($\sim$ms) and ubiquitous in benchmarking but act as weak proxies for true drug viability(Bickerton et al. 2012; Ertl and Schuffenhauer 2009; Gómez-Bombarelli et al. 2018). Pre-trained quantitative structure-activity relationship (QSAR) classifiers, namely DRD2 activity or JNK3/GSK3$\beta$ predictors belong to Tier 2 oracles which are inexpensive and typically slower than Tier 1 heuristics but inherit the biases from the data they are trained on(Olivecrona et al. 2017; Li et al. 2018). Tier 3 oracles include molecular docking dominated by tools namely Glide and AutoDock Vina, operating with explicit target structures at a cost of seconds to minutes per call(Friesner et al. 2004; Trott and Olson 2010). Finally, Tier 4 oracles are the physics-based namely free energy perturbation (FEP), molecular dynamics (MD) and MM-GBSA that offer a higher fidelity at a cost of hours to days, thus prohibitive for the exhaustive RL evaluation pipeline(Cournia et al. 2017; De Vivo et al. 2016; Genheden and Ryde 2015).

##### *Single Objective Reinforcement Learning vs Multi-Objective Reinforcement Learning*

Based on the number of objectives a molecule design model optimizes simultaneously, such models can be divided into two categories: Single-Objective Reinforcement Learning (SORL) and Multi-Objective Reinforcement Learning (MORL)(Zhou et al. 2019). SORL focuses on a single target or objective, unlike MORL which simultaneously optimizes multiple targets based on different requirements, making it more suitable for real world problems. This is achieved by designing special reward functions that combine multiple objectives.

##### *Exploration vs Exploitation*

The trade-off between exploration and exploitation is one of the most crucial aspects a molecular design model must balance. Exploration involves trying alternative actions to discover potentially better rewards, whereas exploitation focuses on selecting actions that are known to yield the highest rewards(Rentschler and Roberts 2025). An optimum exploration-exploitation point is where the model can design molecules with novelty and chemical viability simultaneously.

### 3 Search Methodology

We conducted a systematic literature search following PRISMA-style identification, screening and inclusion strategies for RL-driven molecule design approaches. The pipeline starts by defining primary and secondary keywords to be used for searching in selected databases. Here, we used Web of Science, PubMed, EBSCO and WorldCat databases for the literature search purpose. From the search outputs of each database, we filtered out articles which are irrelevant to our problem statement, review papers or duplicate entries and created an outer join. We also manually included missing

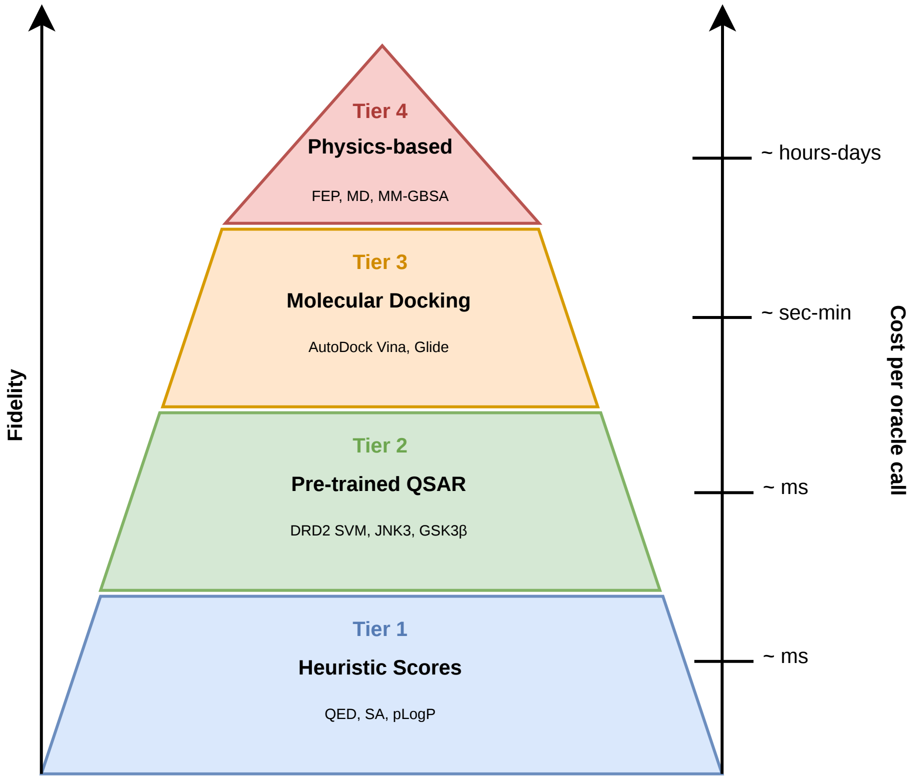

**Fig. 3**: **Oracle tiers for molecular RL rewards.** The fidelity of the oracles are directly proportional to the cost per oracle call. Oracle families can be showcased as a four-tier arrangement starting from heuristic scores (QED, SA, pLogP) as the cheapest followed by pre-trained QSAR models (DRD2 Support Vector Machine (SVM), JNK3/GSK3$\beta$ classifier) and molecular docking oracles (AutoDock Vina, Glide), and finally physics-based oracles (FEP, MD, MM-GBSA) with the top-tier fidelity.

foundational methods published in the domain to the outer join list. Next, another round of filtering is done for the articles which are not exclusively focused on small molecules. We also emphasized the publicly available code bases of the methods as it helps reproduce the findings of the work. Finally, we get a list of 82 articles which we discussed in this review work. Figure 4 shows the end-to-end pipeline of the complete search methodology.

## 4 Taxonomy of RL Algorithms

We organize the survey into four algorithmic families: value-based methods that learn a state-action value function, policy-gradient methods that directly parameterize and update a stochastic policy, reward-proportional methods that sample structures with probability proportional to their reward, and search-based methods that treat molecule optimization as a tree search over the chemical space, and language models form a cross-cutting layer. Section 4.5 treats a further group that is policy-gradient trained, and so belongs to the policy-gradient family above, but is discussed separately because a single action is a tool call rather than a structural edit.

### 4.1 Value-based Approaches

This family updates an explicit value function and remains the smallest branch of the surveyed literature. The family opens with the observation that molecular editing can be framed as a discrete decision problem, in which validity is enforced by construction rather than learned.

As one of the most widely accepted value-based modeling approaches, Zhou et al.(Zhou et al. 2019) combined the domain knowledge of chemistry with double-Q learning in their proposed model named Molecule Deep-Q networks (MolDQN). This approach attempts to mitigate the issue of high variance in gradient estimation of policy gradient-based methods. MolDQN further ensures 100% chemical validity of generated molecules as it modifies a given molecule directly. The authors also emphasized on one of the major limitations of model pre-training in molecular optimization: while pre-training makes it easier to generate molecules similar to the given training set, the exploration ability is limited by the biases present in the training data.

Once Q-learning over molecular edits proved to be viable, the natural next question was whether the same machinery could be pointed at a specific protein rather than at generic property scores. Inspired by the work from Zhou et al., Jeon et al. (Jeon and Kim 2020) proposed a structure-based molecule optimization model named Molecule Optimization by Reinforcement Learning and Docking (MORLD), capable of optimizing lead molecules against a given protein target without prior model training. MolDQN is used inside the RL loop acting as the principal component in the molecule optimization step. In this study, authors used the decaying $\epsilon$-greedy method in which either an action of MolDQN is selected randomly with the probability of $\epsilon$, or the best action according to the current Q function is chosen with the probability of 1-$\epsilon$.

Further research progressed by retaining Q-learning core but shifting the interrogation from how molecules are edited to what the reward is actually measuring. Fang et al.(Fang et al. 2023) highlighted the issue of emphasizing more on diversity and novelty instead of the drug potential of the generated molecules by the current molecular optimization approaches. They therefore proposed a quality assessment-based drug design approach called QADD which integrates an iterative refinement framework with a novel graph-based molecular quality assessment model on drug potentials. A Deep Q-Network (DQN) is used here as it pays attention to the action that maximizes the Q function, regardless of whether the dimension of the action space is fixed or not.

Combined together, these three works trace a single argument: the value-based route buys chemical validity and stable gradients at the price of a hand-specified, discrete action space, and it is precisely that constraint the policy-gradient family sets out to address.

### 4.2 Policy-Gradient Approaches

The majority of RL-driven molecule design approaches fall under this family, spanning foundational frameworks, their direct lineage-based derivatives, and methods organized around a specific classical policy-gradient algorithm.

#### 4.2.1 Foundational Approaches

Williams proposed REINFORCE(Williams 1992), a Monte Carlo policy-gradient method based on the score-function estimator. In molecule generation, a trajectory is typically the stepwise construction of a SMILES string or molecular graph, often associated with a terminal property score. REINFORCE replaces the intractable reward expectation with trajectories sampled from the current policy and uses the realized Monte Carlo return as an unbiased estimate of future reward.

Inspired by REINFORCE, Olivecrona et al. introduced REINVENT(Olivecrona et al. 2017) as a pioneer in developing generative frameworks for the design and optimization of small molecules. The original framework leveraged RNN in combination with RL to generate valid molecules with desirable properties. Later versions of the framework utilized transfer learning (TL)(Blaschke et al. 2020a) and transformer-based approach(Loeffler et al. 2024) for the task. The primary goal of REINVENT was to address the inverse design problem, in which molecular structures must be generated to satisfy predefined chemical properties. Later, a wide array of molecular generation and optimization approaches were built on top of REINVENT.

Continuing with the trend of sequence-based representations of entities, Popova et al. highlighted the scarcity of goal-directed molecule generation approaches and the potential of RL in carving the path of such models as an emerging technology complementary to the established supervised deep neural network-based approaches. They proposed a model named ReLeaSE (Reinforcement Learning for Structural Evolution)(Popova et al. 2018) coupling Stack-RNN with an RL loop in a generator-predictor setup. The objective of Stack-RNN was to learn hidden rules of forming sequences of letters that correspond to legitimate SMILES strings. Being a memory-augmented neural network, Stack-RNN diminished the shortcomings of LSTMs: inability to capture long-term dependencies such as in long SMILES strings. In the RL loop, REINFORCE algorithm was used to maximize the expected reward.

With emergence of GANs in the field of generative modeling, Guimaraes et al. adopted the modeling approach of SeqGAN(Yu et al. 2017) where the data generator was designed as a stochastic policy and further extended the training process towards domain-specific objectives in their proposed approach named ORGAN (Objective-Reinforced Generative Adversarial Networks)(Guimaraes et al. 2017).

Sanchez-Lengeling proposed ORGANIC (Objective-Reinforced Generative Adversarial Network for Inverse-design Chemistry)(Sanchez-Lengeling et al. 2017), a GAN-based chemical compound design approach supported by RL with an application on drug design with desirable properties. They proposed molecule discovery as an inverse-design problem as the objective is to generate molecules given a set of desired properties with an emphasis on efficient chemical space exploration.

Both the sequence-based and adversarial currents, however, inherited the fragility of string representations, which motivated a change in how the molecule is represented while it is being built. You et al. proposed Graph Convolutional Policy Network (GCPN)(You et al. 2018) to address the challenging task of simultaneous optimization of molecular properties and obeying highly complex and non-differentiable physical rules. As one of the pioneers in goal-directed molecule generation, GCPN chose graph representation-based learning as intermediate molecular graphs can act as valid substructures unlike incomplete SMILES representations of molecules that often come out to be chemically invalid. It predicts the action of the bond addition, and is trained via policy gradient to optimize a reward composed of molecular property objectives and adversarial loss.

With the objective of efficient chemical space exploration, Liu et al. proposed a RNN-based de novo molecule generation model called DrugEx(Liu et al. 2019). Later, researchers upgraded DrugEx(Liu et al. 2021) by incorporating the concept of polypharmacology: drug molecules often bind with multiple protein targets resulting in desired or undesired effects. Thus, this was a multi-objective optimization approach, trained with REINFORCE under Pareto-based ranking, to generate drug molecules targeting one desired target and avoiding off-targets. The latest version of DrugEx(Liu et al. 2023) incorporates a Transformer-based architecture that allows scaffold decoration based on the user-provided pre-existing molecule, a feature that was missing in the previous versions.

Collectively these frameworks constructed the working machinery of the field: the generator-predictor decomposition, string versus graph action spaces, learned versus hand-specified rewards, and single- versus multi-objective scoring. The derivative literature below is discussed as a systematic exploration of each of those axes.

#### 4.2.2 REINFORCE-derived Approaches

Here we discuss approaches that adopted REINFORCE as anchor with modifications in the reward, representation, or the search operator it supervises.

Pereira et al.(Pereira et al. 2021a) utilized REINFORCE algorithm in their RL setup with the objective of optimizing molecular structures based on their biological on-target activity and Blood-Brain Barrier (BBB) permeability. The task was designed as an MDP where the actions selected by the agent were the sampling of new molecules by the pre-trained generator. In addition, the external environment evaluating the actions coincided with the evaluation of the molecular properties by the predictor. In another approach, Pereira et al.(Pereira et al. 2021b) adopted classical approach of exploitation and exploration trade-off of the vast chemical space for optimizing molecules with desired properties. Using a generator-predictor couple, the approach shows that with the stabilization of the loss function, generator was able to produce more molecules with desired properties but the diversity in those molecules decreased. Later, Pereira et al.(Pereira et al. 2023b) developed a similar setup with the objective of optimizing molecular structures based on their activity against Ubiquitin-Specific Protease 7 (USP7) protein.

Chadi et al. highlighted the inefficiency of existing molecular design approaches in chemical space exploration and proposed a curiosity-driven approach(Chadi et al.

2023) based on REINFORCE. RNN was used in the generator model which was further fine-tuned to maximize curiosity and desirability. Here, curiosity was defined as the Tanimoto Similarity among the generated molecules.

Hu et al.(Hu et al. 2023a) attempted to solve the problem of pareto optimization of multiple objectives by introducing reward-weighted sum (RWS) that dynamically assigns different weights to molecular properties. REINFORCE was used as the RL component to optimize the generator model to maximize the desired properties of the molecule. Stack-RNN was used to solve the problem of insufficient memory of conventional RNN.

Choi et al. designed COMA(Choi et al. 2023), a model that is capable of optimizing a given molecule in order to optimize its molecular properties. They designed a VAE-based generator to generate structure-constrained molecules with two novel regularization terms of metric learning: contractive and marginal losses. To achieve the goal of optimizing generated molecules that are structurally similar to a given input molecule, but with desired molecular properties, they utilized REINFORCE algorithm.

Mazuz et al.(Mazuz et al. 2023), in a model named Taiga, treated the molecule optimization problem as a language modeling task of predicting the next token using SMILES strings. The objective of this approach was to exploit attention mechanism to overcome the problem of generating invalid SMILES strings, often encountered in RNN-based approaches. REINFORCE was applied as a policy gradient RL for molecular property optimization, complemented by a pretrained transformer model which acts as an initial policy by training on the next-character prediction task. Taiga adopts a two-stage approach: it first learns a general language modeling task of predicting the next token using a GPT-like decoder-based transformer, and then optimizes for the desired properties using RL; its advantage lies in learning both the underlying rules of chemistry and the grammar of SMILES strings.

Fu et al.(Fu et al. 2022) adopted an evolutionary MDP (EMDP) in their proposed approach named Reinforced Genetic Algorithm (RGA). The purpose of the approach was to utilize neural models to profitable design steps for Structure-Based Drug Design (SBDD) and suppress random-walk behavior of the Genetic Algorithm (GA). They adopted REINFORCE to maximize the expected reward as objective, leveraging policy gradient to train a target-ligand policy neural network.

Adury et al. proposed an auto-regressive molecular generation model, SPOTLIGHT, capable of constructing molecules directly at the active site of a given target protein(Adury and Mukherjee 2024). REINFORCE algorithm was patched with GCPN to further tune molecule-level properties directly during the generation phase.

##### 4.2.3 REINVENT-derived Approaches

REINVENT is the most widely adopted molecule design algorithm across the field, and the derivative works below to answer to four distinct questions: how to keep the policy from collapsing, how to make each expensive oracle call count, how to constrain the design task, and how to swap the generator underneath while leaving the loop intact.

Blaschke et al.(Blaschke et al. 2020b) addressed the issue of optimized molecules with low diversity as a result of policy collapse by incorporating a memory unit in the

RL loop of REINVENT. The goal was to modify the reward function with augmented memory in instances where the generative model produces a sufficient number of similar high scoring compounds. This modification forces the model to create new chemical entities that are dissimilar to the original solution to maximize the reward gain, resulting in generation of a library of high diversity compounds.

Fialková et al. proposed LibINVENT(Fialková et al. 2021) which is a scaffold decoration model. It combines an RNN prior, trained with teacher forcing, with REINVENT's augmented-likelihood policy update, which guides the model towards user-defined objectives.

Thomas et al. proposed Augmented Hill-Climb(Thomas et al. 2022) by hybridizing REINVENT with Hill-Climb(Neil et al. 2018) to improve the sample-efficiency and optimization ability. They also used diversity filters with tuned parameters to overcome possible mode collapse. Here, the ultimate goal was to make computationally expensive scoring functions like docking or computer-aided synthesis planning models more accessible on a relevant timescale. The approach uses SMILES molecular representation one-hot encoded into a binary vector as input, and is trained on Maximum Likelihood Estimation (MLE) to predict the conditional probability of the next SMILES symbol based on the prior sequence. Applied to a transformer encoder architecture, character masking allows the transformer to predict the subsequent token by attending over all previously observed symbols rather than using sequence-to-sequence prediction. This generalization to transformer models proves that the RL efficiency gains from the Augmented Hill-Climb strategy extend to other language models.

Sundin et al.(Sundin et al. 2022) approached a major bottleneck in RL-based molecular optimization that is to integrate human feedback in efficient exploration of the vast chemical space. Using REINVENT as the generator, the proposed method is able to learn the scoring function directly from chemist's feedback, replacing the traditional approach of tuning the multi-parameter optimization (MPO) scoring function manually with trial and error. This results in an interactive molecular optimization workflow through the automated exploitation of chemist's knowledge.

Sample efficiency can also be pursued outside the policy update, by making the oracle cheaper to consult. Dodds et al.(Dodds et al. 2024) fused active learning(Sacks et al. 1989; Jones et al. 1998) with REINVENT to improve the sample efficiency of the molecule optimization process by approximating a given oracle function. This RL–AL process consists of two co-dependent loops: in the outer RL loop, REINVENT generates the design space in each iteration, and the inner AL loop searches for the best compounds in this space to assess with the oracle.

Mourdoukoutas et al. proposed ADMETrix(Mourdoukoutas et al. 2025) combining REINVENT with a geometric deep learning architecture for predicting Absorption, Distribution, Metabolism, Excretion, and Toxicity (ADMET) properties called ADMET AI(Swanson et al. 2024). The objective of this approach was to build what authors described as the first ever model to optimize small molecules in real-time across multiple ADMET endpoints.

Wang et al. in their proposed model named Frag-G/M(Wang et al. 2023a) discussed a label-agnostic, multi-constraint molecular optimization approach to mitigate the dependency of large amount of label data of pharmacological and physicochemical properties. REINVENT is used along with a conditional transformer and RNN that resulted in a model capable of generating molecules with 100% validity.

Thomas et al. emphasized on a prompting approach in their proposed model named PromptSMILES(Thomas et al. 2024a). Instead of global optimization of a molecule, the approach dealt with the niche domain of scaffold decoration and fragment linking. Utilizing REINVENT as the RL algorithm, this approach showed that decoder only, SMILES-based chemical language models (CLMs), without retraining, can quickly adapt to task of iterative prompting as well as maintain the ability to optimize arbitrary objectives. CLMs are decoder-only models that are used for their implicitly learned relationships between molecular substructures. In this approach, a molecular substructure (represented using SMILES tokens) is provided as a prompt at inference time to condition the CLM for further molecular generation, which is also used as a constraint for the final molecule to contain that specific substructure. When combined with RL, the CLM adapts to this prompting task and optimizes molecule generation towards different specified objectives.

Rossen et al. proposed RuSH (Reinforced Learning for Unconstrained Scaffold Hopping) (Rossen et al. 2025), a scaffold-hopping model that allows unconstrained, complete molecule generation and optimization using REINVENT. RuSH facilitates designing full molecules having a high three-dimensional and pharmacophore similarity to a reference molecule, but low scaffold similarity, resulting in diverse library compilation.

Zheng et al.(Zheng et al. 2022) elaborated a method to generate PROTACs in a low-resource setting as a novel deep generative model and further optimize generated PROTACs with optimal pharmacokinetic properties using REINVENT.

As a next step in the research from Frag-G/M model, Wang et al. developed 3DSMILES-GPT(Wang et al. 2025a), a 3D molecular generation model that addresses the limitations of the existing molecule optimization approaches that generate molecules with invalid configurations, nonphysical conformations, suboptimal drug-like qualities and limited synthesizability. They adopted a token-only approach that enables the model to comprehensively understand the 2D and 3D characteristics of molecules in large scale. RL approaches adopted from REINVENT was used to further optimize the biophysical and chemical properties of the molecules generated. More recently, they proposed a token-only drug design model capable of encoding both 2D and 3D structural information along with molecular properties into discrete tokens named Token-Mol(Wang et al. 2025b). The model is built on GPT(Radford et al. 2018) and pre-trained with causal masking. Token-Mol introduces a Gaussian cross-entropy loss function tailored for diverse drug design tasks. REINVENT was used to further optimize the pre-trained model parameter $\theta$ so that the optimized model can generate molecules with desired properties.

##### 4.2.4 GCPN-derived Approaches

Khemchandani et al. adopted the principles of graph-based molecular representations from GCPN and proposed DeepGraphMolGen(Khemchandani et al. 2020), a molecular generation method combining molecular graph encoding, RL and multi-objective optimization within a single strategy. Utilizing GCPN in the RL loop for molecule generation, this method introduced a robust loss for experimentally obtained property scores that often have potentially gross errors.

While the preceding work addressed noise in the reward signal, a later effort addressed what the reward should rule out. Zhang et al. used GCPN as backend in their proposed approach named METEOR (Molecular Exploration Through multiplE-Objective Reinforcement) (Zhang et al. 2024c) and emphasized on defining chemistry-based constraints to avoid generating undesired substructures.

#### 4.2.5 PPO-based Approaches

PPO (Proximal Policy Optimization)(Schulman et al. 2017) plays pivotal role in scenarios where trust-region stability is critical. Its clipped surrogate objective limits how far the policy can move in one update, reducing collapse during long SMILES/graph constructions. Approaches based on PPO use discrete token, fragment, or graph-edit actions with sparse terminal rewards.

Simm et al. (Simm et al. 2020) proposed a molecular design framework operating in Cartesian coordinates, incorporating a reward function based on the electronic energy approximated via fast quantum-chemical calculations. Here, the PPO-based policy network is translation and rotation invariant.

Ishitani et al.(Ishitani et al. 2022) formulated the molecular design and optimization problem as a tree-structure construction using deep reinforcement learning (DRL) and proposed a model named RJT-RL utilizing PPO. They propose a novel coarse-grained tree representation of molecules called reversible junction tree (RJT), a unique representation that can be converted reversibly to the original molecule. Superiority of RJT-RL lies in the ease of fine-tuning of the policy and value function models, which is not possible with rule-based fragment growth methods.

Yamada et al.(Yamada and Sugiyama 2023) proposed an interpretable method named MOLDR based on graph decomposition and reassembling to address the challenge of designing molecules with desired properties in the vast candidate space of molecules. Decomposition was carried out by applying frequent subgraph mining to a molecular database to collect a smaller size of subgraphs as building blocks. In the reassembling step, desirable building blocks were searched via PPO and were combined to generate new molecules.

Yue et al.(Yue et al. 2024) addressed the limitations of SMILES-based language models for drug design tasks, namely linker design, due to their autoregressive nature. As a modified version of SMILES, they proposed an unordered fragment-based representation named FU-SMILES. Based on this representation, they trained FragGPT, a universal fragment-based molecular design model. PPO was used to fine-tune the model to generate molecules optimized with multiple biophysical properties. This fragment-by-fragment approach reduces exposure bias and error accumulation.

Ye et al.(Ye 2024) designed the drug design task as a causal language modeling problem. Following OpenAI's ChatGPT and InstructGPT modeling approaches(Christiano et al. 2017), the procedure of reward modeling, supervised fine-tuning, and PPO was utilized to apply transfer learning to a large chemistry model (LCM) for drug design tasks. PPO was used in a feedback loop for optimizing molecular properties such as drug efficacy. The LLM was trained on SMILES or SELFIES

molecular representations, generates molecules token by token, and learns molecular distributions using cross-entropy loss.

Sheikholeslami et al. (Sheikholeslami et al. 2025) addressed the issue of DrugGPT(Li et al. 2023) of generating invalid drug structures for a given target sequence and proposed DrugGen. DrugGen is an LLM, fine-tuned on approved drug-target interactions and optimized with PPO. It incorporates a reward feedback mechanism from protein-ligand binding affinity prediction using pre-trained transformers. Built on the DrugGPT (GPT-2) architecture, DrugGen processes merged strings of protein sequences and SMILES tokens using a BPE (Byte Pair Encoding) tokenizer.

More recently, Javaid et al. proposed AMORTIX(bin Javaid et al. 2026), an amortized Graph Transformer trained with GRPO. GRPO, the group-relative variant of PPO is making waves across the field of RL as it replaces the learned value baseline with a comparison among a sampled group of completions. AMORTIX addresses a structural inefficiency shared by most of the methods surveyed above: an oracle-driven search is restarted from scratch for every new input structure. To mitigate the issue, the learning of AMORTIX was designed as a transferable policy that respects structural constraints supplied at inference time. This design allows optimization of a new starting structure in a single forward pass with no per-molecule search, thus reframing lead optimization from a per-input search problem into an amortized inference problem.

##### 4.2.6 Actor-Critic-based Approaches

Actor-critic methods occupy the middle ground between the two families above, retaining an explicit policy while using a learned value estimate to diminish the variance of its updates.

McNaughton et al. (McNaughton et al. 2022) built 3D-MolGNN$_{RL}$, an autoregressive scaffold decoration model capable of optimizing a core scaffold targeting specific proteins. The goal was to overcome the sequence-based representation of the target and the drug by leveraging a 3D-scaffold-based molecular generation actor over a 2D SMILES generation actor. For the critic model, they utilized parallel graph neural networks as a binding probability predictor to evaluate whether the generated compound actively binds with a target protein.

Chen et al. combined the exploratory capacity of deep generative models and the evolution abilities of genetic algorithms to build MagicMol(Chen et al. 2023). The generator is based on RNN, and represented compound structures as SELFIES to avoid the validity issue associated with SMILES-based approaches. To tackle the mode collapse issue, they utilized truncated reinforcement scores.

Wang et al.(Wang et al. 2023b) emphasized on efficiency and quality in molecular generation process and introduced MomdTDSRL, an approach associated with A2C and heterogeneous parallel supercomputing for large-scale molecular docking based on MPI communication technology. The actor adapts the generation strategy using a transformer-based Deep Neural Network (DNN), with particular emphasis on molecular optimization being incorporated into the reward mechanism. Then, the critic calculates the value of the generation strategy adopted by the actor after receiving the reward.

Park et al. (Park et al. 2025) proposed Mol-AIR leveraging the strengths of both history-based and learning-based intrinsic rewards by exploiting random distillation network and counting-based strategies. An actor-critic-based approach was adopted to efficiently learn intrinsic and extrinsic rewards simultaneously. A dual-critic network was employed to learn both the property-based extrinsic reward and the count-based intrinsic reward. The setup thus aids in efficient chemical space exploration for goal-directed molecular generation.

Kim et al. (Kim et al. 2025a) discussed the inefficiency of autoregressive action-based molecule generation approaches as they often produce invalid molecular substructures due to the lack of explicit optimization feedback. They proposed Synthesizable Hierarchical Action-space Reinforcement learning for Pareto optimization (SHARP), a molecular generation approach that addresses that issue by incorporating a fragment-based hierarchical action space. Soft actor-critic algorithm was used to train a fragment mask generation model that ensures synthetic accessibility of the generated molecules.

Gupta et al. proposed Policy-guided Unbiased REpresentations (PURE)(Gupta et al. 2025) for scaffold constrained molecular generation. The framework combines self-supervised learning and actor-critic algorithm within a molecular transformation environment with reaction rules.

#### 4.2.7 Memory-Augmented and Adversarial Approaches

Cutting across every family above is a shared failure mode: an optimized policy tends to rediscover the same high-scoring molecules. We discuss approaches that address this limitation, either by giving the model an explicit memory of what it has already produced or by applying adversarial reinforcement to the generator.

The earliest attempts coupled both ideas in a single architecture. Putin et al. combined GAN with RL to build RANC (Reinforced Adversarial Neural Computer)(Putin et al. 2018a), one of the earliest approaches in in silico de novo molecule design incorporating a memory module in the architecture. The explicit memory bank was a part of a differentiable neural computer (DNC) with an objective to mitigate the issue of mode collapse in adversarial-based training approaches like ORGANIC. Later, pursuing on the same limitation, they proposed ATNC (Adversarial Threshold Neural Computer)(Putin et al. 2018b) by introducing a novel adversarial threshold block to the RANC model.

Zhavoronkov et al. proposed Generative Tensorial Reinforcement learning (GENTRL)(Zhavoronkov et al. 2019), a deep generative model built for small molecule design and optimization based on the properties. The framework combines variational inference and tensor decompositions into a generative two step machine learning algorithm to discover DDR1 kinase inhibitors.

Guo et al. highlighted that experience replay may aid in improving sample efficiency for molecular generation models. They proposed a novel approach called Augmented Memory(Guo and Schwaller 2024) and introduced a method called Selective Memory Purge that removes entries in the replay buffer with molecular scaffolds undesired for sampling, thus avoiding mode collapse.

#### 4.2.8 General and Hybrid Approaches

The following approaches employ an RL loop without anchoring to any aforementioned algorithms above, either by showcasing the RL components generically or as secondary to a diffusion, VAE, or other generative backbone.

Xiong et al.(Xiong et al. 2023) built a molecule generation model on top of GENTRL based on a GMM (gaussian matrix model) coupled with VAE. The novelty of this approach lies in replacing a self-organized map reward function with a reward derived from a regression model.

Pereira et al. introduced masked language modeling (MLM)(Pereira et al. 2023a) in their RL-driven molecular design approach emphasizing the importance of each atom and functional group in the binding with specific targets, eventually guiding the generator network towards binding-aware molecule generation. The model provides an interpretable approach to address the limitation of existing models that typically condense the evaluation of sampled compounds into a single scalar value, making it difficult for the generative agent to learn the optimal policy.

Researchers show that reward design can also be used to encode medicinal-chemistry knowledge that a docking score is structurally incapable of expressing. Hu et al. proposed an approach called Activity cliff-aware reinforcement learning (ACARL)(Hu et al. 2025), a molecular generation framework that introduces an Activity Cliff Index (ACI) for obtaining the cliff activity of the compounds and a contrastive loss function embedded within RL. The model also highlights the effectiveness of integrating SAR specific knowledge into target-specific molecule design.

Zhang et al. introduced RetroCL(Zhang et al. 2025) highlighting the usefulness of contrastive learning in molecule design. The model learns molecular representations by mapping molecular graphs and local bond features into a latent embedding space using a chemically inspired bond augmentation strategy based on retrosynthesis information.

Xu et al. proposed SGPT-RL(Xu et al. 2024) a framework that employs a generative pretrained transformer (GPT) as the policy network of an RL agent to optimize binding affinity. GPT is trained on lead-like molecules to obtain a prior model that learns the rules of the chemical space, followed by an RL agent that shares the same number of decoder layers as its policy network.

Hu et al. proposed a multi-agent RL framework for molecule design called MolRL-MGPT(Hu et al. 2023b). The model employs multiple GPT-based agents to generate molecules with desired properties while promoting molecular diversity through collaborative exploration of different regions of the chemical space. The framework uses three primary configurations: a prior model pre-trained with unsupervised learning to understand SMILES grammar, a single agent that fine-tunes the prior using RL to optimize docking score and QED scores, and a multi-agent system employing parallel GPT agents with a specialized Encouragement for Diversity (ED) loss function to penalize inter-agent similarity and ensure broad exploration of the chemical space.

Luo et al. focused on generating molecules with effective PROTAC linkers and proposed ProLinker-Generator(Luo et al. 2025). It is another GPT-based molecular generation model combining transfer learning and RL to guide linker generation. It employs transformer blocks with a masked self-attention mechanism to effectively process and generate molecules. Later, Xu et al. proposed SynPROTAC(Xu et al. 2026), that couples reaction-path-driven molecule assembly with RL. In this method, PROTACs are constructed along feasible synthetic routes. A Graph Transformer encodes the warhead or E3 ligand and the model autoregressively samples reactions rather than atoms.

Sheng et al.(Sheng et al. 2025) emphasized on structure constrained molecular generation and proposed a workflow utilizing VAE for pre-training and RL for multi objective optimization of KRAS inhibitors. It introduces several novel evaluation metrics like Knowledge-Integrated Reproduction Score (KIRS) and Composite Diversity Score (CDS) reflecting real-world evaluation. The goal was to optimize drug efficacy and ADMET properties of molecules while focusing on improving BBBp and preserving KRAS inhibitor activity.

Yuan et al. designed a molecular generation framework for 3D structures named MDRL(Yuan et al. 2025), integrating diffusion models and RL for optimizing multi target binding and molecular properties. The diffusion module comprehends the structure of molecules and employs Kolmogorov-Arnold Networks to enhance model performance.

Kumar et al.(Kumar et al. 2026) proposed a two-phase RL framework to design inhibitors for the receptor tyrosine kinase RET. They contrasted two reward regimes directly: potency-focused optimization against a single target, and polypharmacological MPO against resistance mutations.

Guo et al. emphasized that purely target-based rewards overlook system-level cellular response, while phenotype-based screening is experimentally expensive. They proposed XMolRL(Guo and Liu 2026), a model pre-trained on drug-induced transcriptional profiles to address this phenotype-target gap. Fine-tuning is done based on multi-objective RL with composite reward that includes binding affinity and drug-likeness with ranking loss, prior-likelihood regularization and entropy maximization. Following the same transcriptomic route, Matsukiyo et al.(Matsukiyo et al. 2026) built a model on multicontext transcriptome data to simultaneous optimization of drug-likeness, synthetic accessibility and partition coefficient while accounting for system-level biological effects.

Murala et al.(Murala 2026) combined hierarchical variational autoencoders with geometric deep RL for de novo design and activity optimization. The approach emphasized on retaining the 3D structural attributes of the biological entities in the latent space for the policy to act upon.

Wang et al. proposed TSItransRL(Wang et al. 2026a) by pre-training a conditional transformer with multi-property information acting as a teacher and is distilled into a vanilla transformer that is subsequently fine-tuned by RL.

Yadunandan et al. emphasized on a target-agnostic framework that guides generation with reaction templates in their proposed models named ReACT-Drug(Yadunandan and Ghosh 2025). This approach attempted to overcome the dependency of target-specific fine-tuning in the molecule design pipeline.

Every method discussed above carries the same learning signal: increase the expected reward. A policy can be stuck in one high-scoring region of chemical space without requiring to explore any other. The family discussed below explicitly addresses that limitation.

### 4.3 Reward-Proportional Approaches

Bengio et al. introduced GFlowNets(Bengio et al. 2021) to solve the issue of incomplete convergence in a trained policy. The framework fits the setting of a molecular design problem as it casts the construction of a molecule as a sequence of actions over a directed acyclic graph of partial states. Training the forward policy allows the probability of terminating at a specific structure is proportional to its reward rather than concentrated on its maximizer. This approach inspired building more flow-matching based models in future for diverse candidate generation.

Koziarski et al. proposed Reaction-GFlowNet (RGFN) (Koziarski et al. 2024) which generate molecules by chaining basic fragments through established high-yield reactions. Such reactions are drawn from a small library of cheap, accessible building blocks. This allows every sample trajectory to be accompanied by a synthesis route while the reachable space stays orders of magnitude larger than the existing screening libraries.

Cretu et al. persued the same reaction-constrained approach with SynFlowNet(Cretu et al. 2025) where the action space pairs chemical reactions with reactants. This setup allows forward synthesis to acts as an explicit constraint of generation rather than a naive post-hoc filter. Pandey et al. instead relaxed the building blocks with Atomic GFlowNets (A-GFN)(Pandey et al. 2025), constructing molecules atom by atom rather than from predefined fragments to widen the accessible chemical space. Pre-trained on a ZINC subset with inexpensive proxy rewards (drug-likeness, Topological Polar Surface Area (TPSA), synthetic accessibility), the model is adapted to design molecules against specific targets in a goal-conditioned fine-tuning setup.

Instead of working on a limited reaction vocabulary, Seo et al. asked how far that vocabulary can be scaled and proposed RxnFlow(Seo et al. 2025) by training the generative flow over 1.2 million Enamine building blocks and 71 reaction templates. The sub-sampling of the action space occurred in an importance-sampling manner, avoiding the memory inflation of the full space. Later, Kim et took the relaxation further with S3-GFN(Kim et al. 2026) by entirely discarding hard reaction constraints and enforcing synthesizability softly. Here, a sequence-based GFlowNet inherits rich chemical priors from large SMILES corpora. The network is steered by a contrastive auxiliary loss over separate replay buffers of synthesizable and unsynthesizable samples, reweighting probability mass toward the synthesizable region.

Where this family learns a sampler whose terminal distribution matches the reward, the next family dispenses with the learned sampler altogether and searches the space explicitly.

### 4.4 Search-based Approaches

Instead of a gradient-based policy update, the approaches discussed in this section addressed molecule design as a tree search over the chemical space, most commonly via Monte Carlo Tree Search (MCTS).

Yoshizawa et al. proposed ChemTS(Yoshizawa et al. 2022), a MCTS-based molecular optimization model capable of designing molecules against tyrosine kinases while optimizing multiple physicochemical and pharmacological properties. Based on classical generator-predictor architecture, the generator is based on RNN with MCTS algorithm for chemical space exploration, while the predictor module uses Dscore(Winter et al. 2019; Cummins and Bell 2016) as the reward function for simultaneous optimization of 18 objectives.

Iwata et al. further exploited the chemical space search ability of MCTS and proposed VGAE-MCTS(Iwata et al. 2023). While MCTS is able to efficiently search the vast chemical space, it has a tendency to produce chemically invalid molecules. In VGAE-MCTS model, VGAE provides MCTS with a learned, compressed prior over molecule space, allowing limited expansion of search tree starting from a chemically meaningful latent representation.

Erikawa et al. addressed the limitations of SMILES in constructing hit-to-lead design models: generated SMILES are often chemically invalid and it is easier to generate a molecule from scratch using SMILES compared to when the objective is to modify the structure of an existing molecule for enhanced molecular properties. MCTS adds nodes at the end of the tree which is suitable for expanding molecules represented as graphs but not suitable for SMILES. Therefore, they proposed MERMAID (Erikawa et al. 2021) dividing the problem into multiple parts: a) representing each alphanumeric character of a SMILES as a node in the tree, b) extracting independent, chemically valid small subtrees from the complete tree, c) applying MCTS and RNN to add new nodes at the end of the subtree, and d) conjugate the modified subtrees into one valid tree which is scored by a weighted combination of target properties. Later, they proposed Gargoyles(Erikawa et al. 2023), a lead optimization approach capable of optimizing a user-defined molecule, keeping the generated molecules structurally closer to the given molecule but with optimized molecular properties. MCTS was used along with a Graph Convolutional Network (GCN) to develop an autoregressive molecule generation approach with fragment-based tree search.

Nakamura et al. proposed TRACER(Nakamura et al. 2025), which utilizes synthetic pathway generation approach for molecular property optimization. In this approach, MCTS was combined with a conditional transformer and GCN, to optimize compounds targeting specific proteins through the generation of synthetic pathways. Starting from a set of selected reactants as root nodes, MCTS performed direct optimizations of those reactants based on the performed virtual reactions by a transformer pre-trained on a large chemical reactions dataset.

### 4.5 Agentic Approaches

The methods surveyed so far treat the language model, where present, as a policy network that emits tokens and receives a scalar reward. However, an emerging trend can be observed where a general-purpose LLM is utilized as a reasoning agent that acts on a molecule through tools, with RL optimizing the decision sequence.

Yang et al. proposed MolAct(Yang et al. 2025), which formalizes molecular editing and optimization as a sequential tool-guided decision. The training happens in two stages, starting with acquiring editing competence, followed by optimizing properties while reusing the learned editing behavior.

Addressing the recurring sample-efficiency problem, Wang et al. designed MolMem(Wang et al. 2026b) comprising of a long-term memory that accumulates reusable insights across optimization episodes. This approach mitigates the limitation where agents are utilized in a trial-and-error scenario and consume many oracle calls.

Li et al. proposed ChemCRAFT(Li et al. 2026), which decouples chemical reasoning from knowledge storage through agentic RL. This approach allows a small local model to query external chemical knowledge instead of memorizing it, and thereby avoiding both the hallucination of small models and the privacy and inference costs of large cloud-hosted ones.

In summary, these approaches relocate the learning problem from selecting a structural edit to selecting a procedure, shifting the limitation of chemical validity from the policy onto the tools it calls, which aligns closer to how a medicinal chemist reasons about a series.

## 5 Datasets and Benchmarks

In deep learning research, dataset and benchmark are often used interchangeably, but in practice, they deliver different purposes. A dataset is used to train and/or validate the quality of the training procedure of the model, providing an innate scope of the capabilities and limitations of the model. On the other hand, a benchmark, although can hold the schema of a dataset, used to evaluate how better/worse it performs compared to its competitions built for the same task. In this section, we discuss some of the most frequently used datasets and benchmarks for the task of molecule design.

### 5.1 Datasets

This section discusses the frequently used datasets in all the models across the survey majorly for model pre-training, supervised fine tuning and QSAR training.

#### 5.1.1 BindingDB

BindingDB (Liu et al. 2025) is one of the largest public databases of experimentally measured protein-small molecule binding affinities. The affinities are measured in $K_d$, $K_i$, $IC_{50}$ and $EC_{50}$. The latest version (Accessed on: April 16, 2026) of the dataset contains 3.2 million binding measurements for 1.4 million compounds and more than 11,400 targets. The dataset is curated using the data extracted from US patents, ChEMBL and scientific literature. The database is funded by NIH/NIGMS and maintained by UC San Diego.

#### 5.1.2 ChEMBL

ChEMBL (Zdrazil et al. 2024) is a manually curated, open-access, deposited-data aggregation database of bioactive molecules with drug-like properties. As per the latest release (v36: 28 July, 2025), ChEMBL hosts more than 2.8 million unique compounds and approximately 17,800 targets, which account for 24.3 million bioactivity measurements. The data hosted in ChEMBL comes from multiple sources, with PubChem BioAssays and BindingDB Patent Bioactivity being the largest contributors. Being a large bioactivity database, ChEMBL becomes one of the most widely used training data sources for ML/AI drug discovery models, supporting tasks from molecular property prediction to generative chemistry.

#### 5.1.3 ChemCoTDataset

ChemCoTDataset(Hao et al. 2026) is the first chemical reasoning dataset with expert annotated chain-of-thought (CoT) labels. With 22,000 data points, the dataset is meticulously curated to minimize LLM hallucinations on chemical reasoning through supervised fine-tuning (SFT). The dataset thus effectively addresses the reasoning data scarcity issue in chemical reasoning domain for LLMs.

#### 5.1.4 ChemToolDataset

ChemToolDataset(Li et al. 2026) is the first large-scale chemical tool trajectory dataset. The dataset was curated by aggregating raw molecular structures and reaction data from public repositories, including ChEMBL, PubChem and ZINC, based on a novel Tool-Integrated Reasoning Trajectory construction method. The objective was to address the limitation of the traditional method for constructing reasoning dataset by distilling traces from closed-source LLMs, which is static by definition and fails to capture the interactive, hypothesis-driven nature of the modern scientific workflow. This agentic dataset therefore was built on a pure reasoning trajectory with the interaction of a chemical agent toolbox.

#### 5.1.5 CrossDocked2020

CrossDocked2020 (Francoeur et al. 2020) is the first standardized large-scale benchmark dataset for training ML models on cross-docked (non-cognate) protein ligand poses. It is considered as the gold-standard as the standard training/evaluation set for structure-based drug design. It contains 3D structures of ligands docked into similar (but non-cognate) protein binding pockets, with associated pose quality labels (Root Mean Square Deviation (RMSD) to crystal) and binding affinity labels (pK values). The 3D protein structures were collected from protein data bank (PDB), while binding affinity labels (pK) were imported from PDBBind (v2017). CrossDocked2020 hosts 22.5 million poses consisting of 13,839 unique ligands and 2,922 binding pockets. The total number of protein-ligand complexes is 18,450.

#### 5.1.6 ExCAPE-DB

ExCAPE-DB (Sun et al. 2017) is one of the largest publicly available, standardized chemogenomics dataset containing structure-activity relationship (SAR) data. It is curated by merging and standardizing two major public bioactivity databases - ChEMBL (v20) and PubChem. ExCAPE-DB contains appx. 1 million unique compounds and 1,667 targets which account for over 70 million SAR data points, making it useful for building multi-target QSAR models for predictive polypharmacology, off-target effects, and target prediction. ExCAPE-DB became the de-facto data source for several widely used ML benchmarks, including DRD2 oracle in TDC.

#### 5.1.7 GDB

The GDB databases are the largest computationally enumerated collections of theoretically possible drug-like organic molecules. The latest version of the database (v20, 2026) contains a set of 32 trillion molecules within the chemical space of small organic molecules up to 20 atoms of C, N, O, S, F, Cl, Br, and I, following simple chemical stability and synthetic feasibility rules (Buehler et al. 2026). The vast collection of synthetic molecules aids in exploring the theoretical drug-like chemical universe, providing starting points for de novo drug design, fragment-based drug discovery and benchmarking molecular generation models.

#### 5.1.8 GEOM-Drugs

GEOM-Drugs (Axelrod and Gomez-Bombarelli 2022) is the largest publicly available 3D molecular conformer dataset that provides energy-annotated conformer ensembles for drug-like molecules. It is a pioneer in bridging the gap between 2D SMILES datasets (e.g., ChEMBL, ZINC) and physics-based conformer databases. Each of the drug-like molecules in this dataset are annotated with computed conformer energies and statistical weights. GEOM-Drugs contain more than 300,000 unique 3D molecules generated computationally using Conformer-Rotamer Ensemble Sampling Tool (CREST) (Pracht et al. 2020) with GFN2-xTB semi-empirical tight-binding method (Bannwarth et al. 2019). Papers on 3D molecule generation commonly evaluate against GEOM-Drugs, making it the standard benchmark for 3D molecular generative models.

#### 5.1.9 Papyrus

Papyrus is one of the largest publicly available collection of molecules with standardized bioactivity information (Bequignon et al. 2023). The dataset is curated from multiple sources including ChEMBL (v30) and ExCAPE-DB. It contains more than 59 million high-quality compound-protein data points that are comprised of 1.2 million unique compounds and 6,926 proteins. The bioactivity values are measured in units namely $K_i$, $K_d$, $IC_{50}$, $EC_{50}$ (and their logarithmic transforms, e.g. pChEMBL). Unlike earlier datasets, Papyrus comes with quality-annotated bioactivity data points, keeping the load of data pre-processing for training ML models minimal.

#### 5.1.10 PDB

The Protein Data Bank (PDB) (Vallat et al. 2026) is the global archive for biological macromolecular structures, serving as a primary resource for structural biology. The dataset contains three-dimensional atomic coordinates, structure factors, and NMR restraints for macromolecules, as well as detailed crystallization and sequence data. The archive holds more than 250,000 experimentally determined structures, of which roughly 80% were determined by X-ray crystallography, 14% by cryo-electron microscopy, and 6% by NMR spectroscopy.

#### 5.1.11 PDBBind

PDBBind (Wang et al. 2004) is the de facto standard training set for ML scoring functions for binding pose and affinity prediction in drug discovery. It is the only large scale source that systematically links thermodynamic binding data with experimentally evaluated 3D structures. It contains 3D conformations of ligand-protein complexes sourced from Protein Data Bank (PDB). PDBBind is categorized into 3 tiers of quality: a general set, a refined set and a core/test set. General set is the universal set for which all biomolecular complexes for which a binding affinity measurement could be mapped to a PDB structure. Refined set is filtered from general set applying strict structural quality criteria namely resolution, R-factor, ligand completeness and no covalent bonding artifacts. Core set, also called the CASF test set, is the smallest, highest-quality subset, widely used for unbiased benchmarking of scoring functions. The latest version of PDBBind (v2020) contains more than 19,000 complexes in general set, 5,300 complexes in refined set and 285 complexes in core set.

#### 5.1.12 PROTAC-DB

PROTAC-DB (Weng et al. 2021) is the first dedicated database for PROTACs (Proteolysis-Targeting Chimeras). PROTACs are hetero-bifunctional small molecules that exploit the ubiquitin-proteasome system to selectively degrade targeted proteins. Apart from PROTACs, the database integrates structural and experimental data organized around three more core compound classes namely Warheads, E3 ligands and Linkers. Warheads are small-molecule ligands that bind the protein of interest (POI). E3 ligands are also small molecules that recruit an E3 ubiquitin ligase. Linkers are nothing but chemical moieties connecting the warhead and E3 ligand. The latest version of PROTAC-DB (v3, 2025 (Ge et al. 2025)) contains 6,111 PROTACs, 569 warheads, 107 E3 ligands and 2,753 linkers. In addition, authors utilized their inhouse model, PROTAC-Model, to simulate ternary complex structures. The database is in active development phase and the data are manually sourced from other popular databases including PubChem, ChEMBL and BindingDB.

#### 5.1.13 PubChem

PubChem(Kim et al. 2025b) is a primary public chemical database and a foundational data resource for biomedical research, machine learning, and virtual screening. The platform is structured around three central repositories, with Substance holding archived depositor information, Compound capturing distinct chemical structures, and BioAssay containing biological assay data.The dataset contains 119 million unique chemical structures, 322 million substance descriptions, and 295 million bioactivity data points derived from 1.67 million biological assays. Rather than being curated from scratch, PubChem aggregates information from over 1000 diverse sources, including Thieme Chemistry, SpringerMaterials, WIPO, and integrated toxicological data from former ToxNet databases.

##### 5.1.14 QM9

QM9(Ramakrishnan et al. 2014) provides quantum chemical properties for a comprehensive chemical space of small organic molecules, offering a property set of unprecedented size and consistency for benchmarking and developing hybrid quantum mechanics/machine learning models. The dataset characterizes computed geometric, energetic, electronic, and thermodynamic properties including dipole moments, polarizabilities, and enthalpies of atomization . It contains 133,885 stable neutral organic molecules composed of CHONF with up to nine heavy atoms, representing 621 distinct stoichiometries. This collection includes 6,095 constitutional isomers of the predominant stoichiometry ($C_7H_{10}O_2$), for which energetics at the more accurate G4MP2 level are also reported. The dataset was curated as a subset of the GDB-17 chemical universe of 166 billion molecules, with coordinates generated from SMILES strings and refined through iterative quantum chemical geometry relaxations.

##### 5.1.15 USP7

The USP7 (Shen et al. 2023) dataset is a manually constructed in-house library designed for the supervised learning-based identification of Ubiquitin-specific-processing protease 7 (USP7) inhibitors by distinguishing active ligands from inactive ones. Containing more than 4,000 USP7 ligands, this collection is significantly larger than the approximately 2,000 inhibitors previously reported in general scientific literature and patents, making it a robust resource for target-specific modeling. The library characterizes compounds through experimental bioactivity measurements, including $K_i$, $K_d$, and $IC_{50}$ values, and utilizes multimodal molecular representations such as 1D sequential SMILES, physicochemical descriptors, and bit vector fingerprints (MACCS keys and ECFP4). Data was manually curated and aggregated from PubMed, ChEMBL (version 29), and SciFinder, with researchers extracting structural and activity data directly from original publications and using the Open Babel Package for systematic duplicate removal. By providing a comprehensive set of ligands evaluated under three distinct activity cutoffs, the dataset serves as a specialized benchmark for developing high-accuracy virtual screening tools and accelerating drug discovery for cancer therapy.

##### 5.1.16 USPTO

The United States Patent and Trademark Office (USPTO) granted patents hosts around 2 million organic chemical reactions extracted by text-mining from United States patents published between 1976 and September 2016 (Lowe 2017). Later, multiple subsets of the data were made public by various authors. Among those subsets, USPTO-50K, USPTO-MIT (USPTO-480K) and USPTO 1K TPL have been widely used in several ML-driven drug discovery tasks. USPTO-50K (Schneider et al. 2015) is one of the earliest subsets created with 50,000 chemical reactions data. It is the most widely used benchmark for retro-synthesis prediction. USPTO-MIT (USPTO-480K) (Jin et al. 2017) is the largest subset among all, with appx 480,000 chemical reactions. Due to the large size, it became the standard large-scale benchmark for forward (product) prediction. USPTO-1K TPL (Schwaller et al. 2021) is a relatively new subset that contains information for appx 450,000 chemical reactions with 1,000 template labels (hence the name 1K TPL). This subset was designed for fine-grained reaction classification. All these subsets contain SMILES representations of reagents participating in various chemical reactions.

##### 5.1.17 ZINC

ZINC15(Sterling and Irwin 2015) is a comprehensive, open-access database and suite of chemoinformatics tools designed to bridge the gap between chemical libraries and biological research by providing a ready to dock collection of commercially available compounds. The dataset contains over 120 million purchasable drug like molecules representing nearly all organic molecules currently for sale and hosts a total of 230 million searchable compounds. The library is curated by aggregating data from 122 annotated catalogs supplied by over 266 vendors, with each compound annotated by its biological target, gene association, and physical properties such as molecular weight and lipophilicity ($\log P$). By organizing molecules into specific subsets based on their chemical properties and commercial availability, ZINC15 enables researchers to rapidly identify and procure lead-like candidates for virtual screening and experimental validation. Figure 5 showcases the frequency of datasets used by the approaches across the survey.

#### 5.2 Benchmarks

While a dataset determines what a model can learn, a benchmark determines the extent of that learning compared to other models. The benchmarks below are some of the most frequently utilized across the surveyed literature.

##### 5.2.1 MOSES

MOSES (Molecular Sets)(Polykovskiy et al. 2020) measures unconditional generation quality, i.e., how well a model reproduces a curated, drug-like chemical space (e.g., ZINC-derived, filtered for Molecular Weight (MW) and SA score). The benchmark contains about 1.59M training molecules and 176K test molecules, with MW within the range of 250–350, at most 7 rotatable bonds and XlogP $\leq 3.5$.

##### 5.2.2 GuacaMol

GuacaMol(Brown et al. 2019) is one of the widely used de novo drug generation benchmarks, used in assessing whether an algorithm can generate chemically plausible molecules from scratch. It comprises two orthogonal suites: a) distribution-learning benchmarks to assess how well a generator reproduces the training-set distribution, and b) goal-directed benchmarks encompassing 20 tasks testing property optimization.

##### 5.2.3 PMO

PMO(Gao et al. 2022) emphasizes on sample efficiency for molecular optimization under a fixed oracle-call budget. It is comprised of 23 oracle functions covering essential single-property objectives (e.g., QED, penalized logP, JNK3, GSK3$\beta$, DRD2) and multi-property composite tasks.

#### 5.2.4 TDC

Therapeutic Data Commons (TDC)(Huang et al. 2021) is an enriched platform for AI-ready datasets, task definitions, evaluation tools, leaderboards, and data-processing utilities spanning different domains in drug discovery. For drug design, it provides property oracles such as predicted activity, docking-related objectives, and synthesizability-oriented criteria.

#### 5.2.5 DOCKSTRING

Dockstring(García-Ortegón et al. 2022) is a compilation of standardized docking-based benchmarks for regression, virtual screening, and de novo design using AutoDock Vina wrapped in a fully automated, reproducible pipeline. It contains 260,155 diverse drug-like molecules docked against 58 medically relevant targets, resulting in $\geq$15M docking scores and poses.

#### 5.2.6 MolScore

MolScore(Thomas et al. 2024b) is a unified, modular framework for scoring, evaluating, and benchmarking generative models for de novo drug design. It allows assembling the evaluation protocol using modular components, including molecular similarity objectives, docking-based scores, and physicochemical property filters.

#### 5.2.7 ChemCoTBench

ChemCoTBench(Hao et al. 2026) is a reasoning framework that formalizes chemical problem-solving into transparent, step-by-step workflows. It addresses the limitations of current chemical reasoning benchmarks that focus on simple knowledge retrieval and neglect step-by-step reasoning required for complex tasks such as molecular optimization and reaction prediction. ChemCoTBench formulates complex chemical tasks into explicit sequences of verifiable modular chemical operations on SMILES structures, allowing the assessment of an LLM's ability to execute fundamental chemical transformations. The benchmark contains 1,495 samples across 22 chemical tasks.

Aforementioned benchmarks appear to measure the same thing at different levels of fidelity. Nevertheless, these benchmarks can be segregated across three axes: whether the benchmark scores a distribution or a search (MOSES, GuacaMol-DL), whether the oracle budget is capped (GuacaMol-GD, PMO, Dockstring), and what kind of oracle is permitted at all (TDC, MolScore). ChemCoTBench is the only benchmark in this set that evaluates a transformation of a given input rather than the molecules generated by a model unconditionally. Table 2 showcases the comparative scenario of the benchmarks discussed in this work. In the table, "Lead input?" denotes whether the benchmark scores a modification of a user-supplied starting molecule rather than molecules the model produces unprompted.

**Table 2**: Comparison of benchmarks used across the surveyed literature.

| Benchmark | Primary Focus | Oracle Budget | Main Metric | Docking? | Synthesizability? | Target-Specific? | Lead input? |
|---|---|---|---|---|---|---|---|
| MOSES | Distribution learning | None | FCD, Validity, IntDiv | × | SA score filter | × | × |
| GuacaMol-DL | Distribution learning | None | FCD, Kullback-Leibler (KL) divergence | × | × | × | × |
| GuacaMol-GD | Goal-directed | Unlimited | Normalized score | × | × | Partial | × |
| PMO | Sample efficiency | 10,000 | AUC Top-10 | × (heuristic) | × | Partial | × |
| TDC | Multi-task + oracles | Configurable | Task-specific | ✓ (Vina/Glide) | × | ✓ | × |
| DOCKSTRING | Docking quality | 5,000 | Top-10 docking score | ✓ (Vina) | × | ✓ (58 targets) | × |
| MolScore | Modular scoring | Tracked | AUC, IntDiv, Scaffolds | ✓ | ✓ | ✓ | × |
| ChemCoTBench | Editing + optimization | n/a (single-shot) | Pass@1, ΔProp, SR% | × | × | Partial | ✓ |

The benchmarks discussed above are separated based on oracles and budgets instead of the metrics they evaluate, which are not mutually exclusive: validity, diversity and top-$K$ scores are reported across most of the surveyed benchmarks, with exceptions for Pass@1 and ΔProp (ChemCoTBench) which expects a supplied input molecule. Table 3 lists the major metrics along with their brief description and range information.

**Table 3**: Evaluation metrics reported across the surveyed benchmarks.

| **Metric** | **Definition** | **Typical range** |
|---|---|---|
| Validity | % SMILES parseable by RDKit | 0–1 |
| Uniqueness | % non-duplicate among generated | 0–1 |
| Novelty | % not in training set | 0–1 |
| FCD | Fréchet distance in ChemNet activation space | 0–$\infty$ (lower = better) |
| IntDiv | Avg. pairwise Tanimoto distance ($1 -$ sim) | 0–1 |
| AUC Top-K | Area under top-$K$ avg. score vs. oracle calls | 0–1 |
| SA score | Synthetic accessibility (fragment-based) | 1–10 (lower = easier) |
| QED | Quantitative estimate of druglikeness | 0–1 |
| Docking score | AutoDock Vina / Glide predicted binding $\Delta G$ | kcal/mol (lower = better) |
| Diverse Top-K | Avg. reward of $K$ molecules with pairwise Tan. distance $>$ threshold | Oracle-dependent |
| EF (Enrichment Factor) | Rate of actives in top-$K$ / baseline rate | 1–$\infty$ (higher = better) |
| Top-K avg. score | Average oracle score of best $K$ molecules found | Oracle-dependent |
| Pass@1 | % of single attempts yielding a valid molecule that satisfies the requested edit | 0–1 |
| ΔProp | Mean change in the target property between input and output molecule | Oracle-dependent (signed) |
| SR% (Success rate) | % of instances where ΔProp exceeds a task-specific threshold | 0–1 |

## 6 Case Study: Lead optimization against DRD2

This case study aims to optimize the QED, SA score and DRD2 activity of selected leads and an FDA-approved DRD2 antagonist, risperidone. QED and SA are two of the fundamental heuristic oracles to estimate the drug-likeness of a compound. On the other hand, DRD2 activity is a Tier 2 oracle and one of the most widely used benchmark objectives in RL-driven molecule design.

We selected four lead compounds: 1-phenyl-4-propylpiperazine (Lead#1), 4-(4-chlorophenyl)-4-hydroxypiperidine (Lead#2), 3-(4-propylpiperazin-1-yl)phenol (Lead#3) and 3-(piperazin-1-yl)benzonitrile (Lead#4) to be optimized in this case study. The rationale behind designing such diverse list of leads was to analyze if the seeds of those leads are able to probe the range of situations the lead optimization model will actually face. A single seed cannot distinguish a method that genuinely optimizes from one that merely reports whatever a good starting point already is.

All the selected lead compounds are built on the canonical DRD2 orthosteric pharmacophore: an aromatic ring joined to a basic, protonatable nitrogen held in a six-membered saturated N-heterocycle. Such an arrangement is responsible for the formation of salt bridge to Asp114(3.32), while the aromatic contact defines dopamine receptor recognition(Durdagi et al. 2016). Each of the lead compounds therefore is a deconstructed, validated DRD2 recognition core which is small enough to grow into potential drug-like compounds. The molecules are of low molecular weight (187-220 Da, i.e. large mass headroom under drug-like limits), synthetically simple (SA 1.5-2.3), and drug-like (QED 0.71-0.84).

Risperidone is an FDA-approved atypical antipsychotic and a potent antagonist of the DRD2 receptor(Wang et al. 2018). Its structural properties satisfy both Lipinski(Lipinski et al. 2001) and Veber(Veber et al. 2002) rules and thus holds very less potential to be optimized further. In practice, risperidone is expected to resist structural modification, making it the negative control defining the reward ceiling. Table 6 shows the SMILES representations and Figure 6 showcases the physicochemical properties of all the five compounds selected for the case study.

#### *Experimental Setup*

We compare two models for the case study: TRACER(Nakamura et al. 2025) and RGFN(Koziarski et al. 2024). TRACER is based on MCTS over a reaction-template expansion with a learned transformer policy, while RGFN is a reaction-based GFlowNet trained with trajectory balance, which samples in proportion to reward. Each of the methods is run five times per lead with different random seeds. Comparisons are made at matched oracle budget: each method's output is truncated at the number of distinct molecules the other scored. Both methods elaborate molecules through a fixed library of reactions or templates, and neither library covers all five leads.

Scaffold coverage is a property of the reaction library a method carries, irrespective of its search algorithm. Risperidone defeats both libraries, while Lead#1 defeats RGFN's. We report this as a shared limitation of reaction-constrained generation, and restrict the quantitative comparison to the three leads both methods can address.

#### *Results and Observations*

Figure 7 shows the optimization trajectories and the per-run outcomes behind Table 5. As per those outcomes, the two models succeed in different axes. TRACER reaches the higher peak composite on every lead it addresses (0.902 vs. 0.870 on Lead#2; 0.902 vs. 0.887 on Lead#4). Template-constrained MCTS is an efficient hill-climber in a lead's immediate neighbouhood. RGFN returns substantially more of the space above the lead: on Lead#2, $\sim 3\times$ the seed-beating molecules in $\sim 4\times$ the distinct modes at identical oracle spend. Pooling five runs, TRACER returns 11 modes on Lead#2 and 15 on Lead#4, exactly what a single replicate returns, while RGFN's runs pool to 87 and 36 modes respectively, roughly twice any single run.

**Table 4**: Lead coverage of the two reaction libraries.

| Starting lead | TRACER (templates) | RGFN (133 anchored reactions) |
|---|---|---|
| Lead#1 | elaborated | **0 applicable reactions — not addressable** |
| Lead#2 | elaborated | 24 applicable |
| Lead#3 | elaborated | 1 applicable |
| Lead#4 | elaborated | 25 applicable |
| Risperidone | **no analogues generated** | **0 applicable reactions — not addressable** |

Neither method optimizes a lead that starts near the reward ceiling. Lead#3 begins at composite 0.884 and TRACER exceeds it by 0.009 with exactly one molecule. RGFN, with a single applicable reaction, never exceeds it. Reported improvement on this lead therefore should not be considered as optimization.

A trade-off of ceiling for breadth can be observed for unseeded generations. RGFN run in its published de novo setting on the same reward reaches $0.843 \pm 0.027$ with the highest top-100 internal diversity of any arm (0.574), below the anchored ceiling but exploring a wider region.

**Table 5**: Lead-optimization results at matched oracle budget (mean ± sd over five runs). "Modes" are greedy Tanimoto-0.5 cluster representatives among molecules scoring above the starting lead (Morgan $r$=2, 2048 bits); "modes pooled" re-clusters the union of all five runs. $\text{IntDiv}_{100}$ is the mean pairwise Tanimoto distance over the top-100 molecules.

| Lead (seed) | Method | Oracle calls | Best | # > seed | Modes/run | Modes pooled | $\text{IntDiv}_{100}$ |
|---|---|---|---|---|---|---|---|
| Lead#2 (0.626) | TRACER | 2952 | $0.902 \pm 0.000$ | $229 \pm 0$ | $11.0 \pm 0.0$ | 11 | 0.453 |
| | RGFN | 2952 | $0.870 \pm 0.018$ | $650 \pm 440$ | $42.8 \pm 20.1$ | 87 | 0.502 |
| Lead#3 (0.884) | TRACER | 1936 | $0.893 \pm 0.000$ | $1 \pm 0$ | $1.0 \pm 0.0$ | 1 | 0.502 |
| | RGFN | 1936 | $0.688 \pm 0.000$ | $0 \pm 0$ | $0.0 \pm 0.0$ | 0 | 0.434 |
| Lead#4 (0.611) | TRACER | 1467 | $0.902 \pm 0.000$ | $353 \pm 31$ | $15.0 \pm 0.0$ | 15 | 0.495 |
| | RGFN | 1467 | $0.887 \pm 0.012$ | $225 \pm 68$ | $14.8 \pm 3.9$ | 36 | 0.474 |

The case study establishes that on one target with one composite objective and two reaction libraries, the reward-maximizing and reward-proportional families are not ordered. The first returns better single molecules, while the later returns more of such molecules and does not repeat itself across runs. What the case does not establish is a general ranking: one target, one QSAR oracle, no synthesis or binding validation, and no wall-clock comparison.

## 7 Challenges and Limitations

Despite reported success in RL-driven molecular optimization strategies, several challenges persist that are needed to be addressed.

Amidst these challenges, mode (or policy) collapse is the most frequently reported one that arises mainly when the RL agent consistently converges onto the narrow set of high scoring structures and got trapped in local optima rather than exploring diverse regions of the chemical space. Attempted mitigation include incorporating scaffold-memory and diversity filters that penalize repeated Bemis–Murcko scaffolds, as discussed in memory-assisted REINVENT. Mol-AIR also addressed the issue by utilizing intrinsic-curiosity rewards that explicitly reward exploration of novel scaffolds. Nevertheless, a consensus metric defining "sufficient" diversity in a drug-discovery context is non-existent. The reward-proportional family addresses this failure at the level of the objective, as a trained GFlowNet terminates at a structure with probability proportional to its reward.

An RL agent inherits the biases of the pre-trained generative prior it fine-tunes and of the training data. This results in dilemma for the agent between staying close enough to the prior to generate synthetically valid, drug-like molecules and exploring far enough in the chemical space to discover novel structures. For example, a prior trained on a particular chemical library with KL regularization essentially prevents the policy from drifting into invalid regions but also confines the agent to the prior's distribution and hinders exploration.

Reward hacking is the phenomenon where an agent exploits the weaknesses associated with a low-fidelity oracle and maximizes the predicted score by drifting into regions where the predictor is unreliable. The agent thus repeatedly generates molecules with high scores and is prevented from discovering genuinely better molecules.

Majority of RL-approaches avoid defining feasible synthesis routes for generated molecules. Low-fidelity, post-hoc oracles such as SA score are easy to hack, thus a low nominal SA score does not guarantee synthetic tractability. The issue is addressed by reaction- and fragment-constrained methods namely TRACER that restrict the action space to feasible transformations, but at the cost of a more restricted search space.

Sample efficiency benchmarks such as PMO show that trivial genetic algorithm baselines can match the degree of sampling of RL methods under constrained budgets. Several state-of-the-art RL approaches require thousands of oracle calls to converge, while majority of the approaches degrade sharply under realistic wet-lab budget of $\leq 500$ evaluation cycles.

While benchmarks for de novo molecule generation are abundant, benchmarks that specifically evaluate optimization of a user-supplied lead molecule are comparatively scarce, and those that exist, such as ChemCoTBench, rely on cheap heuristic oracles that do not reflect true drug viability. DOCKSTRING demonstrated that methods showcasing state-of-the-art performance on low-fidelity objectives can fail on high-fidelity docking objectives, underscoring how sensitive reported performance is to the choice of benchmark.

The modern agentic approaches relocate chemical competence from the policy weights onto external tools. This eliminates the invalid-SMILES failure mode but replaces it with a hallucinated intermediate that yields a parseable and plausible molecule that no validity filter will reject.

Finally, unavailability of wet-lab validations for the curated molecules undermines the practicality of the predictions. Systematic synthesis-assay-validation pipelines are needed to be integrated into the workflow as a mandatory post-generation step to showcase the potential these methods in generating viable drugs as proof rather than mere promise.

## 8 Discussions and Future Scope

RL for molecular optimization has evolved from proof-of-concept SMILES generators to the most recent reaction-aware and language-model-guided pipelines over the last decade. In this survey, we highlight several robust themes and open directions as prospective approaches. Firstly, failure to balance exploration against exploitation by existing RL-based approaches has been a recurring theme across the survey, highly correlated with the phenomena like mode collapse and prior bias. Future studies thus must emphasize on better structured, diversity-aware exploration and on reward signals that favor true molecular novelty over proxy-score maxima.

Search-based (MCTS) and reward-proportional (GFlowNet) methods have converged on the same objective from different directions, both constraining the action space to feasible reactions instead of scoring synthesizability post-hoc. This allows every terminal structure to carry a route. Nevertheless, they differ in their learning schema. For MCTS, the search itself resolves the exploration-exploitation trade-off, resulting in no learning that persists between inputs. A GFlowNet learns a sampler whose terminal distribution matches the reward, as showcased in the case study. The two families thus should be read as complementary, with reward-proportional sampling to enumerate regions above a lead, and a reward-maximizing search to refine within them.

Replacing SMILES-based representation of molecules with SELFIES can solve the issue of generating syntactically invalid molecules in the intermediate or final steps of the RL loop. Unlike SMILES, every SELFIES token is derived formally from a well-structured grammar, thus every string produced by any combination of tokens decodes to a chemically valid molecule. Such a representation therefore will allow the reward signal to focus on optimizing other objectives.

A prominent shift can be observed from RNN- or Graph Neural Network (GNN)-based generators toward transformer-based chemical language models (CLMs) as the policy network for RL fine-tuning. Starting from GPT-inspired decoder-only generators to large chemistry models fine-tuned with PPO, all these approaches demonstrate efficient reward modeling for successful molecular design. LLM prompting-based methods further show that pre-trained CLMs can be used post-hoc for scaffold decoration or fragment linking without retraining, demonstrating the in-context adaptability of such models. Recent approaches extend this shift one step further by replacing the token-emitting policy with a general-purpose reasoner that calls chemical tools, which decouples chemical competence from the policy weights. Nevertheless, such a shift leaves the field without a benchmark that scores tool-call trajectories under a capped oracle budget.

## Declarations

### Author Contribution

**K.D:** Data Curation, Conceptualization, Methodology, Writing - Original Draft Preparation, Writing - Review & Editing, Visualization. **T.S:** Formal Analysis, Investigation, Writing - Original Draft Preparation, Writing - Review & Editing. **P.G:** Conceptualization, Resources, Writing - Review & Editing, Project Administration, Supervision, Funding Acquisition.

### Funding

This work was partially supported by CHRB-2360623 (PI: Das), NSF-2316003 (PI: Ghosh), NIH 1S10OD038214-01 (PI: Ghosh), NIH 1T32TR006000-01 (PI: Cifu), NIH 1R01AR086251-01A1 (PI: Boyan), VCU Breakthrough (PI: Ghosh) and VCU CCTR Endowment funds (PI: Thangarajh) awarded to P.G.

### Competing Interests

The authors declare that they have no conflict of interest regarding the publication of this paper.

### Ethics Approval and Consent to Participate

Not applicable. This study did not involve human participants, human data, or animals.

### Consent for Publication

Not applicable.

### Data Availability

Code and data generated for the case study is available at https://github.com/debnathk/drd2_case_study.

**Table 6**: List of leads optimized using TRACER along with Risperidone

| Lead | SMILES |
|---|---|
| Lead#1 (1-phenyl-4-propylpiperazine) | `CCCN1CCN(c2ccccc2)CC1` |
| Lead#2 (4-(4-Cl-phenyl)-4-OH-piperidine) | `OC1(c2ccc(Cl)cc2)CCNCC1` |
| Lead#3 (3-(4-propylpiperazinyl)phenol) | `CCCN1CCN(c2cccc(O)c2)CC1` |
| Lead#4 (3-piperazinyl-benzonitrile) | `N#Cc1cccc(N2CCNCC2)c1` |
| Risperidone | `CC1=C(C(=O)N2CCCCC2=N1)CCN3CCC(CC3)-C4=NOC5=C4C=CC(=C5)F` |

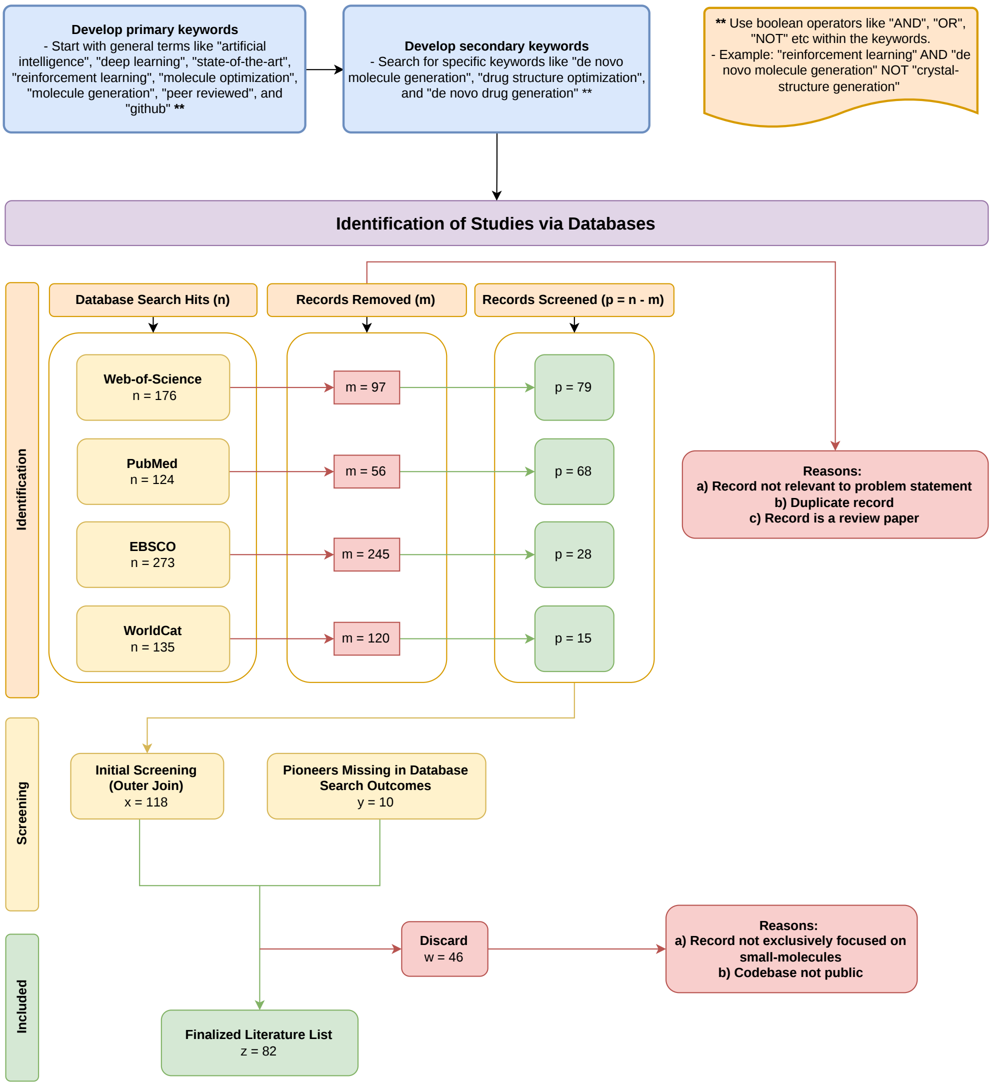

**Fig. 4**: **End-to-End Search Methodology.** Keywords are constructed prior to querying the databases. Primary corpus is developed based on unique entries of relevant and original methodology articles. The secondary corpus is the concatenation of the outer join of the primary corpus and manually included state-of-the-art methodology articles missing the corpus. Secondary screening is done based on the focus on small molecule-only methods and availability of the universally accessible code base, resulting in a final list of 82 articles.

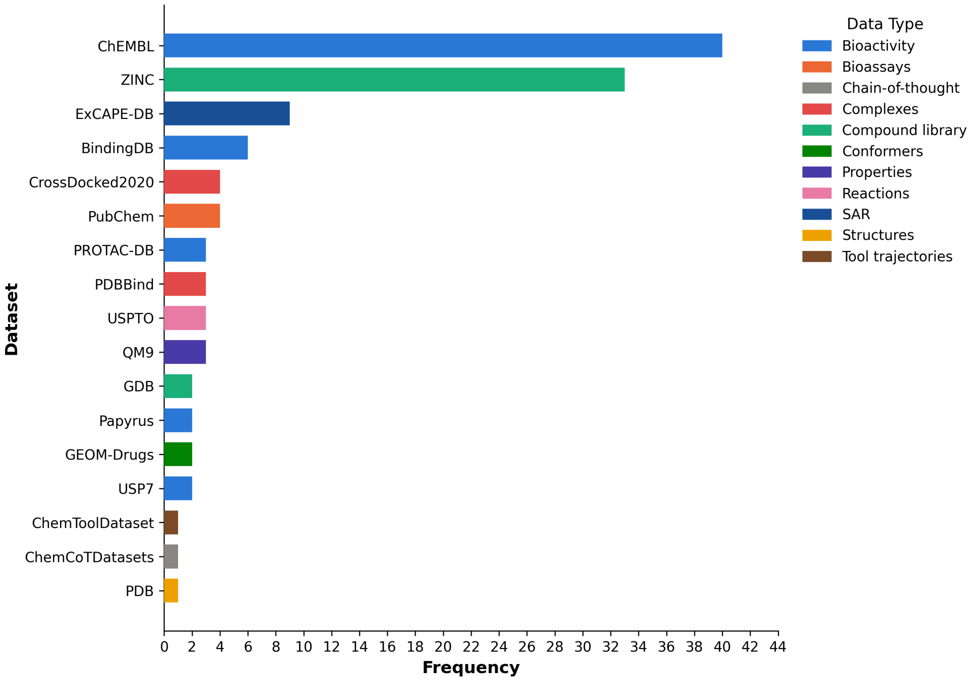

**Fig. 5**: **Dataset distribution across the survey.** ChEMBL and ZINC are the most frequently used datasets for training.

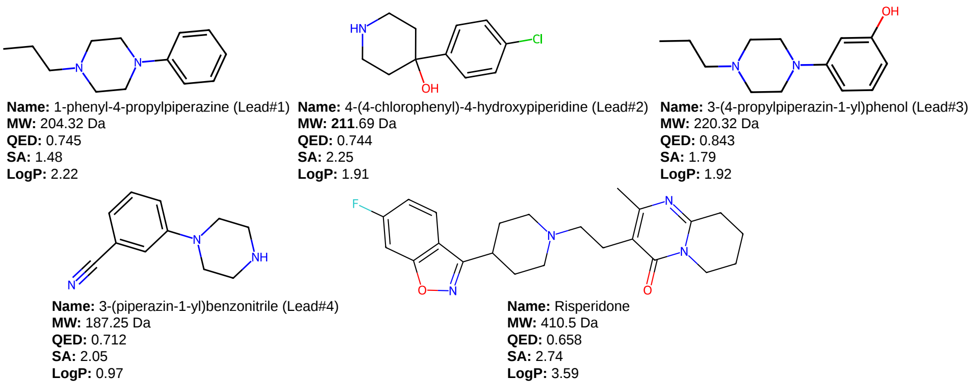

**Fig. 6**: **Selected leads for optimization case study.** Molecular structure of the four leads along with risperidone with physicochemical properties is shown.

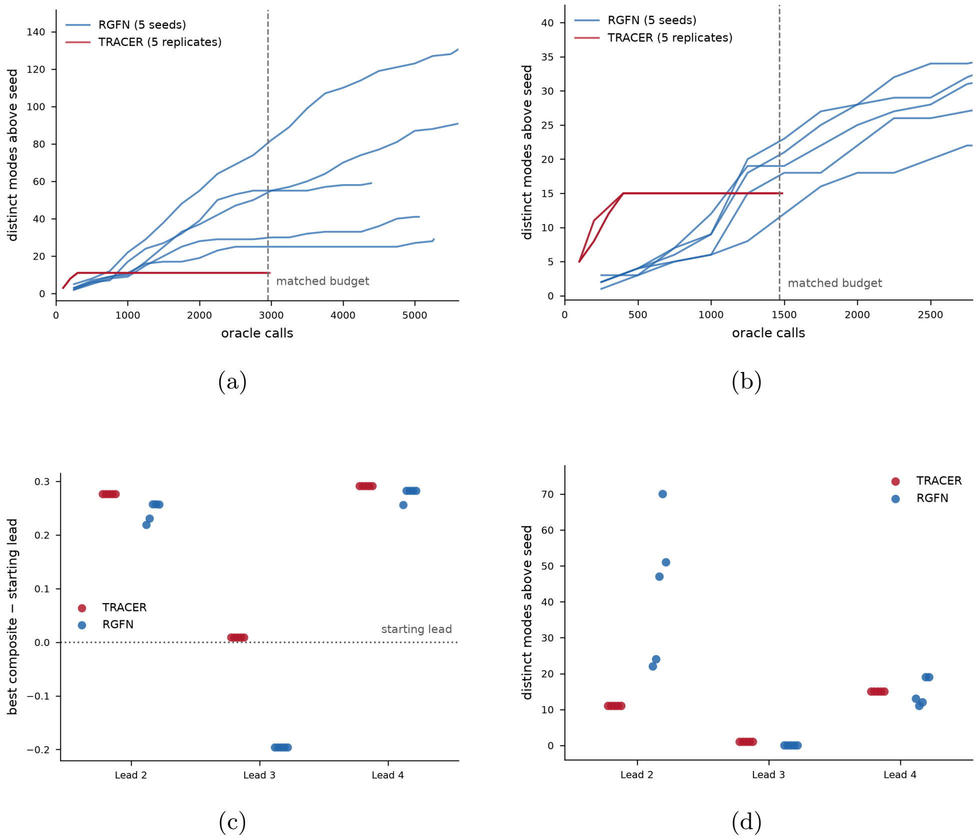

**Fig. 7**: **Outcomes of the lead-optimization case study.** (a, b) Distinct modes above the starting lead against oracle calls, five runs per method, for Lead#2 and Lead#4; the dashed line is the matched-budget cut. TRACER's five traces coincide. (c) Peak composite improvement over the starting lead per run at matched budget. (d) Run-to-run spread in mode count at matched budget.
